# The Role of LVV‐H7 in Alcohol‐Induced Reward Mechanisms

**DOI:** 10.1111/adb.70086

**Published:** 2025-09-05

**Authors:** Przemyslaw Mielczarek, Kinga Hartman, Eagle Yi‐Kung Huang, Ewa Gibula‐Tarlowska, Pawel Grochecki, Tymoteusz Slowik, Jolanta H. Kotlinska, Jerzy Silberring, Anna Drabik

**Affiliations:** ^1^ Faculty of Materials Science and Ceramics AGH University of Krakow Krakow Poland; ^2^ Laboratory of Proteomics and Mass Spectrometry, Maj Institute of Pharmacology Polish Academy of Sciences Krakow Poland; ^3^ Department of Pharmacology National Defense Medical Center Taipei Taiwan; ^4^ Graduate Institute of Medical Sciences National Defense Medical Center Taipei Taiwan; ^5^ Department of Pharmacology and Pharmacodynamics, Faculty of Pharmacy with Division of Medical Analytics Medical University of Lublin Lublin Poland; ^6^ Experimental Medicine Center Medical University of Lublin Lublin Poland

**Keywords:** addiction, alcohol, alcohol‐related reward, binding targets, hemorphins, LVV‐H7

## Abstract

This study aimed to investigate the role of LVV‐hemorphin‐7 (LVV‐H7) in alcohol dependence. LVV‐H7 is a short peptide derived from the cleavage of haemoglobin chains that binds to opioid receptors and plays diverse roles in various physiological and pathological processes. Additionally, LVV‐H7 is cleaved at higher concentrations in the presence of alcohol. We conducted behavioural experiments in animal models and performed proteomic analyses of CNS tissues from alcohol‐addicted rats to identify LVV‐H7 binding partners. Using fluorescent microscopy, we confirmed the blood–brain barrier (BBB) permeability of synthesized LVV‐H7 and its releasing enzyme inhibitor, pepstatin. Our results revealed a dose‐dependent correlation between LVV‐H7 quantities and alcohol levels. Mass spectrometry‐based analyses identified LVV‐H7's protein‐binding targets in CNS tissues of addicted rats and the enzymes responsible for their degradation. These findings highlight the significant role of LVV‐H7 in the mechanisms underlying alcohol dependence and indicate the potential role of hemorphin as a therapeutic target.

## Graphical abstract

This study explores the role of LVV‐H7, a hemorphin peptide, in alcohol dependence by examining its interaction with the opioid and NMDA receptor systems. Behavioral analysis revealed that LVV‐H7 enhanced ethanol‐induced conditioned place preference at lower alcohol doses, suggesting its role in alcohol reward. Proteomic analysis identified LVV‐H7‐binding partners and degradation pathways, implicating Puromycin‐Sensitive Aminopeptidase (PSA) in hemorphin metabolism. These findings suggest that LVV‐H7 modulation could serve as a potential therapeutic target for alcohol use disorder.

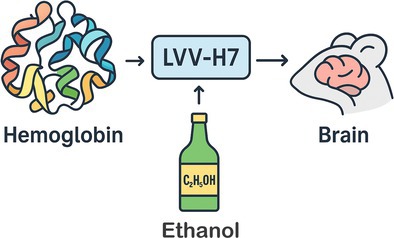

## 1 Introduction

Alcohol dependence is a chronic disorder marked by compulsive alcohol consumption, withdrawal symptoms and a high risk of relapse. The neurobiological mechanisms underlying alcohol dependence involve a complex interplay of neurotransmitters, neuromodulators, endogenous opioid peptides and transcriptome [1, 2]. Among these, hemorphins—a family of bioactive peptides derived from haemoglobin have gained attention for their potential role in alcohol‐related behaviours and neurophysiological changes [3]. This essay examines the involvement of hemorphins in alcohol dependence, their interactions with the opioid system, and their broader implications in the pathophysiology of alcohol use disorder (AUD).

Hemorphins are endogenous peptides derived from the enzymatic degradation of haemoglobin. These peptides exhibit opioid‐like activity by binding to opioid receptors, particularly the mu‐opioid receptor (MOR), which plays a central role in reward and addiction [4]. Hemorphins also modulate other neurotransmitter systems, such as dopamine and gamma‐aminobutyric acid (GABA), both of which are implicated in alcohol dependence. The opioid system is crucial for mediating the reinforcing effects of alcohol. Alcohol consumption stimulates the release of endogenous opioids, activating MORs in the mesolimbic reward pathway, which enhances dopamine release in the nucleus accumbens. This process contributes to the pleasurable effects of alcohol and reinforces drinking behaviour.

The tetrapeptide hemorphin‐4 (H4), a fragment of the human haemoglobin β‐chain (residues 34–37), contains the Tyr‐Pro‐Trp‐Thr sequence, which is crucial for opioid‐like activity in all hemorphins. Several enzymes, including pepsin [5], lysosomal proteases such as cathepsin D [6] and aspartic endoproteases [7], have been implicated in the generation of hemorphin.

The first endogenous hemorphin, LVV‐H6, was identified from human pituitary tissue using peptide sequencing [8]. Subsequent mass spectrometry analyses of hemorphins in cerebrospinal fluid (CSF) were conducted [9, 10]. Studies on hemorphin distribution and metabolic stability have also been conducted in rat brain tissue [11]. Additionally, research has explored the enzymatic pathways involved in hemorphin generation and the possible mechanisms of opioid peptide cleavage [12]. The effects of various incubation conditions, solvent composition, pH [7] and ethanol on hemorphin stability have also been examined [13]. However, precise data on hemorphin‐degrading enzymes remain lacking, and no specific hemorphin fragments have been definitively identified. Instead, only predictions based on enzymatic action mechanisms have been suggested [14, 15]. Notably, in vitro studies indicate that alcohol stimulates LVV‐H7 formation [13, 16].

**FIGURE 1:**
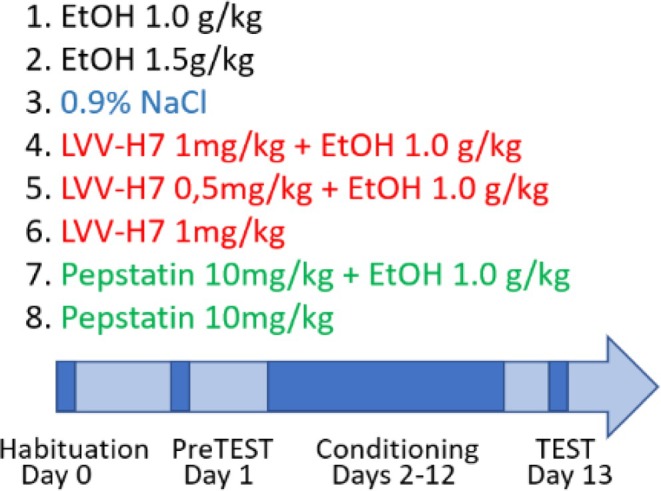
Schematic representation of experimental groups and administered substances.

In this study, we explored the in vivo role of the cryptide LVV‐H7 in alcohol dependence using proteomic approaches. Synthetic LVV‐H7 was administered in an animal model, and behavioural assessments were performed across eight experimental groups (Figure 1) using the conditioned place preference (CPP) paradigm. Animals received intraperitoneal injections of alcohol, LVV‐H7, pepstatin (a cathepsin D inhibitor that blocks LVV‐H7 cleavage from the haemoglobin β‐chain), NaCl, or combinations of alcohol with LVV‐H7 and pepstatin.

We assessed changes in protein expression in brain regions associated with alcohol dependence, focusing on the MOR, NMDA receptor, protein phosphatase DARPP‐32 and angiotensin‐converting enzyme (ACE). Immunoassays quantified proteins indirectly interacting with LVV‐H7 in the mesolimbic–mesocortical dopaminergic system and spinal cord across all experimental groups. Protein binding targets of LVV‐H7 were identified via a pull‐down assay using hemorphin‐conjugated magnetic beads to elucidate potential mechanisms of action.

Furthermore, we quantified LVV‐H7‐degrading enzyme activity using specific substrates for cathepsin D, a known LVV‐H7‐cleaving enzyme. These experiments were performed in vivo (via pepstatin injection) and in vitro using CNS structures, including mesolimbic–mesocortical brain regions and spinal cord tissue from tested animals.

## 2 Materials and Methods

### 2.1 Animals

All conducted experiments used male Wistar rats (Experimental Medicine Center, Lublin, Poland) weighing 220 ± 20 g. The animals were housed under standard laboratory conditions (22°C, 12:12‐h light–dark cycle; light on at 8:00 AM) in groups of four rats per cage. The animals were allowed 7 days for acclimation before experiments with access to standard food (Bacutil; Motycz, Poland) and water ad libitum. After 7 days of adaptation and handling, the animals were divided into groups of 7–8 and prepared for the tests. Behavioural experiments were carried out according to the National Institute of Health Guidelines for the Care and Use of Laboratory Animals and the European Community Council Directive for Care and Use of Laboratory Animals (86/609/EEC). The Local Ethics Committee approved them. Additionally, we confirm that ARRIVE guidelines report the study.

### 2.2 Substances

LVV‐H7 was dissolved in 0.9% NaCl and administered intraperitoneally (i.p.) 15 min before experiments at doses of 0.5 and 1 mg/kg. Control groups received 0.9% NaCl injections in an equivalent volume and by the same route. Pepstatin (Sigma‐Aldrich) was dissolved in 0.9% NaCl and administered i.p. at 10 mg/kg. Ethanol (95% w/v, Polmos; Poznan, Poland) was diluted with 0.9% NaCl to a 15% w/v concentration. In the CPP procedure, ethanol at doses of 1 or 1.5 g/kg (15% w/v) was administered i.p. before each conditioning phase.

### 2.3 CPP Apparatus

In our experiments, eight identical rectangular CPP boxes (60 cm × 35 cm × 30 cm), each one composed of three compartments: two large compartments (25 cm × 35 cm) and a smaller central grey compartment (10 cm × 10 cm), were used. In one of the compartments, the inside walls were white, and the floor held a large grid, whereas the walls of the other compartment were painted black, and the floor held a narrow grid. Illumination in the testing room was adjusted so that the environmental (visual and tactile) cues would not produce a significant baseline preference for a specific compartment. Both large compartments were separated from a central grey area by the removable guillotine doors, the opening of which provided access to the larger compartments. The apparatus was thoroughly cleaned between each test procedure and then wiped with dry paper towels to neutralize odour traces. The boxes were kept in a soundproof room masking neutral noise and a dim 40‐lx light. The CPP performance was measured by computerized video tracking.

### 2.4 CPP Procedure

The CPP paradigm was carried out according to an unbiased procedure [17, 18, 19] with minor modifications. The CPP procedure lasted for 13 consecutive days and consisted of the following phases: a habituation (1 day), a pre‐test (1 day), a conditioning (10 days) and a test (1 day) as described below. The time spent in each compartment was measured during the pre‐test phase. These results separated animals into groups with approximately equal biases for each side. Moreover, an appropriate control group (0.9% NaCl‐treated during all phases of experiments) underwent the same CPP procedure as the drug‐treated rats.

On the first day of testing (habituation phase), all rats were placed in the CPP apparatus's central grey square compartment and allowed to explore both conditioning compartments freely for 30 min.

During the second phase (pre‐conditioning test), 24 h after the habituation session, rats were placed in the central grey square compartment of the CPP apparatus with free access to all compartments for 15 min. The time spent in each compartment was recorded with a computer programme. No injections were given to rats during these first two sessions.

During the third phase (conditioning), beginning 24 h later, rats were conditioned once daily for 10 consecutive days with ethanol (1 or 1.5 g/kg, 15% w/v) or the same volume of 0.9% NaCl. The rats were continuously injected immediately before being placed into the conditioning chamber, and the guillotine doors, which separate the central grey area from the two compartments, were closed.

During each conditioning day, rats were conditioned with one vehicle and drug‐environment pairing separated by at least 6 h. Groups were counterbalanced for drug order (morning or evening), drug side and drug chamber association. This procedure was chosen to avoid circadian (morning/evening) variability.

The CPP (expression phase) test was conducted approximately 24 h after the last conditioning session. Rats were given free access to the experimental compartments for 15 min, during which the amount of time spent in each of the two large compartments was recorded as described above for the pre‐conditioning test. No injections were given to rats during this phase.

### 2.5 The Effect of LVV‐H7 on the Acquisition of Ethanol‐CPP

During the experiment's first phase, the rats were conditioned once daily for 10 consecutive days, either with ethanol (1 or 1.5 g/kg, 15% w/v) or the same volume of 0.9% NaCl. This procedure was carried out to select the non‐rewarding dose of ethanol used in the following experiment. To determine the effects of LVV‐H7 on the non‐effective ethanol dose in the CPP, LVV‐H7 (0.5 and 1 mg/kg; i.p.) or 0.9% NaCl were given 15 min before each injection of 0.9% NaCl or ethanol (1 g/kg, 20% w/v) during the 10‐day conditioning phase (acquisition period). On the expression day, no injection was given, and the rats were allowed free access to all compartments of the apparatus in a drug‐free state for 15 min. Moreover, a separate group of rats was evaluated in the above CPP paradigm without ethanol administration to rule out the possibility that LVV‐H7 administration alone may evoke rewarding or aversive effects in the CPP test. All animals were treated with 0.9% NaCl or LVV‐H7 (0.5 and 1 mg/kg; i.p.) 15 min before conditioning. The rats were trained as described above by receiving counterbalanced administration of one of these agents and 0.9% NaCl. On the expression day, the rats were given free access to the apparatus for 15 min, and the time spent in each compartment was measured.

### 2.6 The Effect of Pepstatin on the Expression of Ethanol‐CPP

To determine the effects of pepstatin on the ethanol‐CPP, pepstatin (10 mg/kg; i.p.) or 0.9% NaCl was given 5 min before each injection of 0.9% NaCl or ethanol (1.5 g/kg, 15% w/v) during the 10‐day conditioning phase (acquisition period). On the expression day, no injection was given, and the rats were given free access to all compartments of the apparatus in a drug‐free state for 15 min. A separate group of rats was evaluated in the above CPP paradigm without ethanol administration to rule out the possibility that pepstatin alone may evoke rewarding or aversive effects in the CPP test. All animals were treated with 0.9% NaCl or pepstatin (10 mg/kg; i.p.) 5 min before the conditioning. The rats were trained as described above by receiving counterbalanced administration of one of these agents and 0.9% NaCl. On the expression day, the rats were given free access to the apparatus for 15 min, and the time spent in each compartment was measured.

### 2.7 CPP Statistical Analysis

Data collected in the CPP test were expressed as means ± SEM of the preference scores (i.e., the differences between post‐conditioning and pre‐conditioning time spent in the drug‐paired compartment). One‐way analysis of variance (ANOVA) with Tukey's post‐test was used to analyse the effect of ethanol doses on the occurrence of the rewarding effect of ethanol in the CPP test. A two‐way ANOVA was used to determine the impact of LVV‐H7 or pepstatin (treatment), pretreatment (ethanol vs. 0.9% NaCl) or the interaction between these factors, followed by the Bonferroni test to compare each group with the control group. Statistical significance was set at *p* < 0.05. All statistical analyses were performed using the GraphPad Prism 6.0 software.

### 2.8 Peptide Synthesis Reagents

The TentaGel S AC Phe‐Fmoc resin was purchased from Rapp Polymere. Other chemicals used in the synthesis, i.e., Fmoc‐protected amino acids, (1‐Cyano‐2‐ethoxy‐2‐oxoethylidenaminooxy)dimethylamino‐morpholino‐carbenium hexafluorophosphate (COMU), *N,N*‐diisopropylethylamine (DIPEA), *N,N*‐dimethylformamide (DMF), dichloromethane (DCM), trifluoroacetic acid (TFA), phenol, triisopropylsilane (TIS), acetaldehyde, chloranil, diethyl ether and piperidine, ammonium bicarbonate, formic acid, acetonitrile, dithiothreitol, iodoacetamide and α‐cyano‐4‐hydroxycinnamic acid were from Sigma‐Aldrich. Trypsin Gold, Mass Spectrometry Grade was obtained from Promega.

### 2.9 Synthesis of LVV‐hemorphin‐7 (LVV‐H7)

Synthesis of LVV‐H7 was performed manually using standard 9‐fluorenylmethoxycarbonyl (Fmoc) solid phase synthesis. The peptide was assembled on the TentaGel S AC Phe‐Fmoc resin, which has a capacity of 0.23 mmol/g. Couplings were performed with a threefold excess of Fmoc‐amino acids, a threefold excess of COMU, and a sixfold excess of DIPEA in DMF as a coupling medium. The couplings were allowed to proceed for 2 h at room temperature. Then, the resin was washed (3xDMF, 3xDCM, 2xDMF) to remove excess reagents. Fmoc protecting groups were removed using 20% piperidine in DMF. The resin was washed as described above. Coupling efficiency and deprotection steps were confirmed using the chloranil test. Upon completion of the chain assembly, the peptide was cleaved from the resin with TFA:phenol:TIS:water (88/5/2/5, v/v/v/v) for 2 h. The resulting peptide was precipitated from diethyl ether and dried under vacuum. The high‐resolution MS characterized the product, and preparative HPLC assessed its purity.

### 2.10 Synthesis of LVV‐H7 Immobilized on the Resin

**FIGURE 2:**
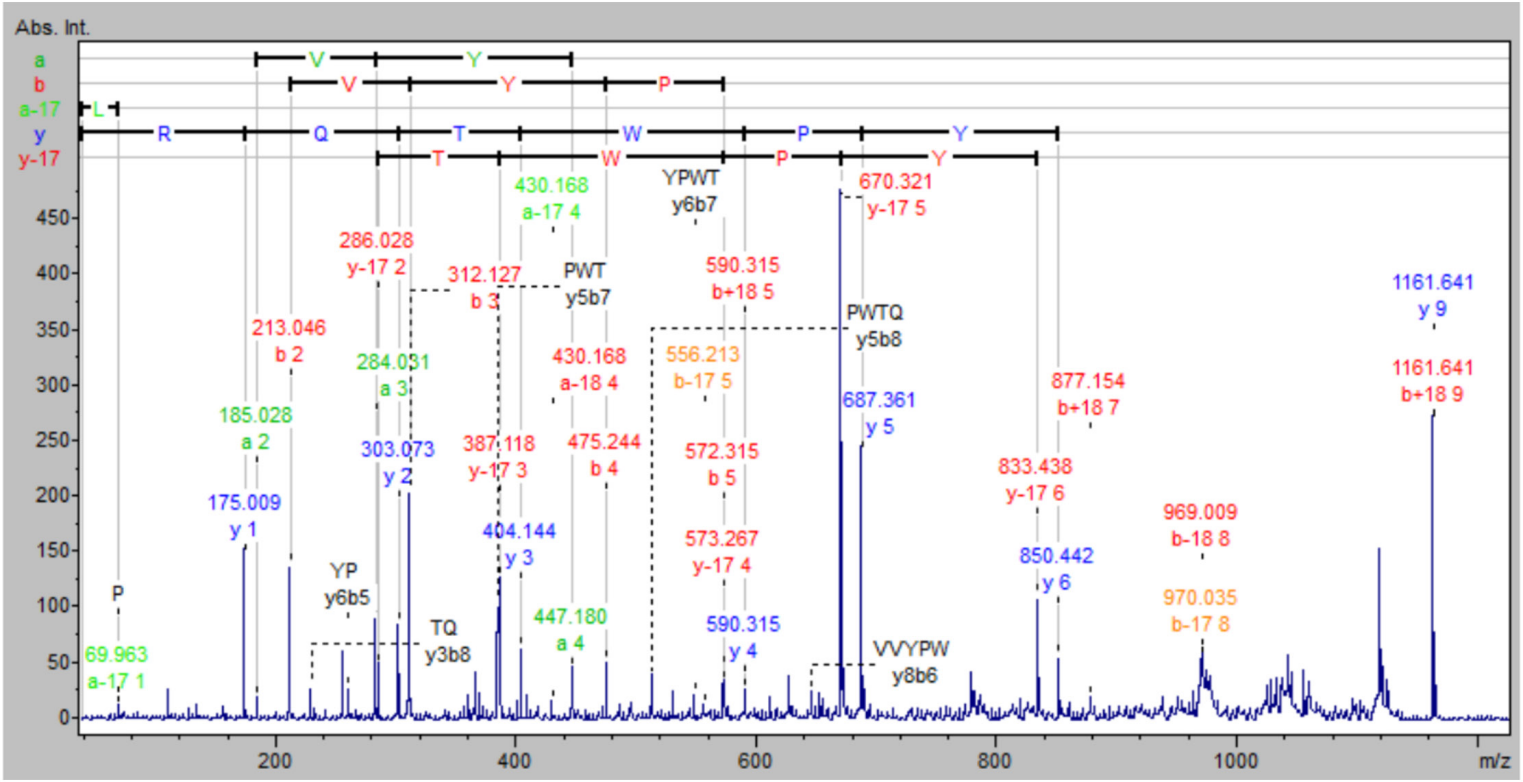
Fragmentation spectra for the ion 1161.6 m/z showing the amino acid sequence of the enzymatic digest of peptidyl resin (Leu‐Val‐Val‐Tyr‐Pro‐Trp‐Thr‐Gln‐Arg). The sequence was assigned using the BioTools software from Bruker Daltonics.

Immobilization of hemorphin on the solid support was performed using the Tentagel S NH_2_ resin. The synthesis was done to optimize the linker length by attaching one, two, and three molecules of 6‐aminohexanoic acid, respectively. Subsequently, the Fmoc group was removed, and the protected amino acids were consecutively coupled, following the procedure described above. Upon completion of the synthesis, the α‐amino groups of N‐terminal amino acid residues were deprotected. The resin was then dried overnight under vacuum, and the side‐chain protecting groups were cleaved off for 2 h with the mixture of TFA/TIS/H2O (95:2.5:2.5; v:v:v). The resin was then rinsed twice with methanol and dried under vacuum. The peptidyl‐resin was stored at 4°C for further analysis. The peptidyl resin was subjected to enzymatic digestion by trypsin, using the following protocol to confirm the sequence of the obtained peptide. The resin with deposited LVV‐H7 was washed with 100 mmol/L ammonium bicarbonate for 3 h. After centrifugation and removal of the supernatant, an enzymatic reaction with trypsin was carried out (2 pmol of trypsin on each 10 mg of peptidyl resin was used). The reaction was carried out at 37°C for 1 h. The solution was analysed by the MALDI‐TOF/TOF using α‐amino‐4‐hydroxycinnamic acid (HCCA) as a matrix, and the results are presented in Figure 2.

### 2.11 Synthesis of Pepstatin and Hemorphin Labelled With 5(6)‐Carboxyfluorescein

The fluorescent analogue of pepstatin was synthesized on the Tentagel S RAM resin. At first, the Fmoc‐Lys (Mtt)‐OH was introduced using COMU/DIPEA, according to the procedure described above. To remove the Mtt group, the dried resin was treated with the TFE/HFIP/TIS/DCM mixture: 1:2:0.5:6.5 (v/v). Labelling with 5(6)‐carboxyfluorescein was performed with 5(6)‐carboxyfluorescein/HOBt/DIC in a molar ratio of 1:1:1. The couplings were allowed to proceed for 2 h. The following steps involved Fmoc removal and coupling of amino acids, using standard procedure and conditions, as described above. Treatment of the resulting peptidyl resin with cleavage cocktail (containing TFA) has led to the peptide cleavage of the resin. The peptide was then precipitated from diethyl ether and dried under vacuum. The high‐resolution MS characterized the probe, and preparative HPLC assessed its purity.

### 2.12 Enzyme Assay

Three spinal cords were homogenized in 0.6 mL of Tris–HCl buffer (pH 7.4) for 1 min each, followed by centrifugation (25 000 ×*g*, 20 min at 4°C). According to the manufacturer's protocol, protein concentration was measured using the Bradford method (Sigma‐Aldrich). The extract was applied directly on a HiPrep DEAE FF 16/10 column and eluted with a linear gradient of NaCl (0–1 M) in 20 mM Tris–HCl buffer, pH 7.8. Fractions of 5 mL were collected at a flow rate of 5 mL/min. The enzymatic activity was measured at pH 7.4 (Tris–HCl) using 10 μL of each fraction and 10 μL of LVV‐hemorphin (60 μg/mL) as a substrate in a total volume of 40 μL. The reaction was performed at 37°C for 1 h, mixing at 500 RPM. To terminate the reaction, ice‐cold methanol was added to each tube. The enzyme activity was monitored by measuring the formation of fragments cleaved from LVV‐H7 using mass spectrometry. Fractions with enzymatic activity were concentrated using the Amicon Ultra‐2 3 kD and prepared for further MS analysis.

The probes (50 μL each) were incubated with 75 μL of 50 mM DTT in 50 mM NaHCO_3_ at 90°C with mixing at 500 RPM. Subsequently, the reduced proteins were carbamidomethylated by adding 75 μL of 100 mM iodoacetamide and incubated at 37°C with mixing at 500 RPM. Finally, trypsin (5 ug) in 150 μL of 50 mM NH_4_HCO_3_ was added, and the mixtures were incubated overnight at 37°C with mixing at 500 RPM. After completion of digestion, the probes were desalted using the Pierce C18 spin columns and analysed by MS.

The nanoLC‐MS/MS analyses were performed using the Easy‐nLC II nanocapillary chromatography system (Bruker Daltonics, Germany), as published earlier [20, 21]. Separating peptides was performed using a 3 μm Biosphere C18 column (10 cm long, 75 μm ID, 3 μm particle size, NanoSeparations, Nieuwkoop, the Netherlands). The gradient was formed using Phase A: 0.1% formic acid in water and Phase B: 0.1% formic acid in acetonitrile at a total flow rate equal to 300 nL/min. The system was controlled by the Hystar software (Bruker Daltonics, Germany). The gradient was maintained from 2% to 45% of Phase B in 30 min, then 90% of Phase B for 10 min, and again reduced to 2% at 60 min for column equilibration.

Fractions eluted from the column were directly mixed with a matrix and deposited on a 384 MALDI target plate using the Proteineer fc II sample collector (Bruker Daltonics, Germany). Fifteen‐second fractions were collected, and 96 fractions for one sample, Αlpha‐cyano‐4‐hydroxycinnamic acid, were used as a MALDI matrix. The mass spectrometry analyses were performed on the Ultraflextreme (Bruker Daltonics, Germany) in positive ion mode.

The acquired mass spectra and fragment mass spectra were analysed using FlexAnalysis software (Bruker Daltonics, Germany). They were processed using the Mascot algorithm (Matrix Science version) against the Swiss‐Prot database. The search parameters were set: taxonomy: all entries; fixed modifications: carbamidomethyl; variable modifications: methionine and oxidation; allowed for up to 1 missed cleavage; peptide charge: +1; mass tolerance: 25 ppm for precursor mass. Proteins with the Mascot score higher than 30 and with the level of false positives *p* ≤ 0.05 were considered identified.

### 2.13 Binding Partners

Rat brains, three from controls and three from ethanol‐dependent rats, were homogenized in 5 mL of Tris–HCl buffer (pH 7.4) for 1 min each. Homogenates were centrifuged for 10 min at 500*g* at 4°C. The supernatants were collected and centrifuged for 20 min at 25 000*g* at 4°C. According to the producer's protocol, protein concentration was measured using the Bradford method (Sigma‐Aldrich). The reaction was performed in the Eppendorf tubes. Ten milligrams of peptidyl‐resin was placed in each tube and activated by washing in methanol and phosphate‐buffered saline (PBS). The appropriate volume of homogenate (10 mg of protein for each probe) was then added and incubated at 4°C for 30 min. After the completion of the reaction, the probes were centrifuged at 1000*g* at 4°C, and the supernatant was removed. The resin was rinsed twice with PBS solution and then subjected to alkylation, reduction, and digestion, according to the above procedure. The samples were further desalted using Pierce C18 spin columns and analysed by MS using the abovementioned method [22].

### 2.14 Western‐Blotting

Brain structures and spinal cords isolated from the animals were homogenized in 50 μL of the buffer consisting of 50 mM Tris/HCl, pH 7.6 and 150 mM NaCl in the presence of a proteinase inhibitor cocktail (Roche). Cells were homogenized thrice on ice by sonication (5 s each). The homogenate was centrifuged at 25 000*g* for 30 min at 4°C. The final protein concentration in the supernatant was determined using a Bradford assay kit (Sigma‐Aldrich).

One‐dimensional polyacrylamide gel electrophoresis was performed after standardizing the samples according to their total protein concentration. Thirty microlitres of the Laemmli sample buffer (Bio‐Rad, USA) were added to the subsequent volume of each sample, followed by incubation for 5 min at 90°C in a Thermomixer (Eppendorf). According to the Bio‐Rad protocol, proteins were separated on the 10% SDS‐PAGE.

Mini Protean system and electrotransferred onto the PVDF membrane (Bio‐Rad, USA). The adequate antibodies confirmed the presence of proteins (Biorbyt, UK). The blotted membranes were treated with casein solution (Vector Laboratories, USA) as a blocking agent for 45 min at room temperature, washed three times with Tris‐buffered saline (TBS; Sigma‐Aldrich), and subsequently incubated with antibody for 1 h, according to the manufacturer's recommendations (NMDAR1 orb99445, ACE1 orb107454, OPRM1 orb216172, DARPP‐32 orb106530). After extensive washing, the blots were incubated with the horseradish peroxidase‐conjugated goat anti‐rabbit IgG secondary antibody (65‐6120, Thermo, USA) for 1 h with TBS supplemented with 0.1% Tween 20. Finally, the blots were visualized using the One‐step TMB solution (Thermo, USA). Densitometric analysis was performed using the GelDoc XR+ (BioRad, USA) according to the previously published method [23].

## 3 Results

### 3.1 Synthesis of LVV‐H7

The solid phase synthesis prepared the peptides using the standard Fmoc (9‐fluorenylomethoxycarbonyl) strategy, according to the following procedure [24, 25]. Optimizing the linker length was performed to assess the potential impact of steric hindrance of hemorphin immobilized on the solid support. The mass spectrum recorded for the mixture obtained after incubation of the synthesized peptidyl‐resins with trypsin is presented in Figure 2. Based on the obtained results, it can be concluded that the amount of Ahx acid residues does not significantly affect the accessibility of the substrate and the rate of enzymatic digestion of LVV‐H7 peptide immobilized on the TentaGel resin. The amino acid sequence of the peptidyl resin digestion product was tested through tandem MS. The trypsin digest's correct sequence confirmed the LVV‐H7 peptide synthesis accuracy on a solid support.

### 3.2 Vascular Permeability of Synthetic Hemorphin and Pepstatin

**FIGURE 3:**
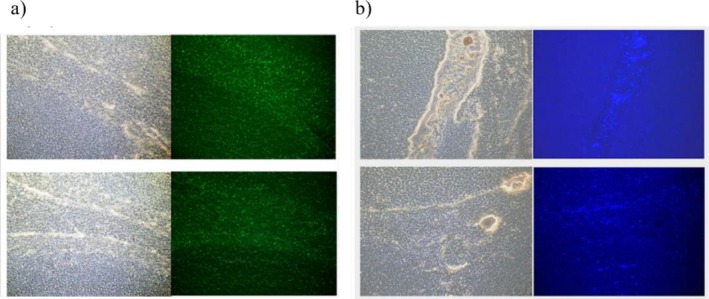
Rat brain sections under visible light and fluorescence microscopy 10×: (a) pepstatin labelled with carboxyfluorescein, (b) LVV‐H7 labelled with Dansyl.

We investigated the ability of synthesized LVV‐H7 and pepstatin to cross the blood–brain barrier (BBB). Fluorescently labelled compounds—pepstatin conjugated with carboxyfluorescein and LVV‐H7 tagged with Dansyl—were administered i.p. to rats according to Section 2.2. Brain tissue samples from treated rats were then analysed using fluorescence microscopy. Our results demonstrate that both LVV‐H7 and pepstatin successfully cross the BBB (Figure 3).

### 3.3 Effect of LVV‐H7 on the Acquisition of Ethanol‐CPP

**FIGURE 4:**
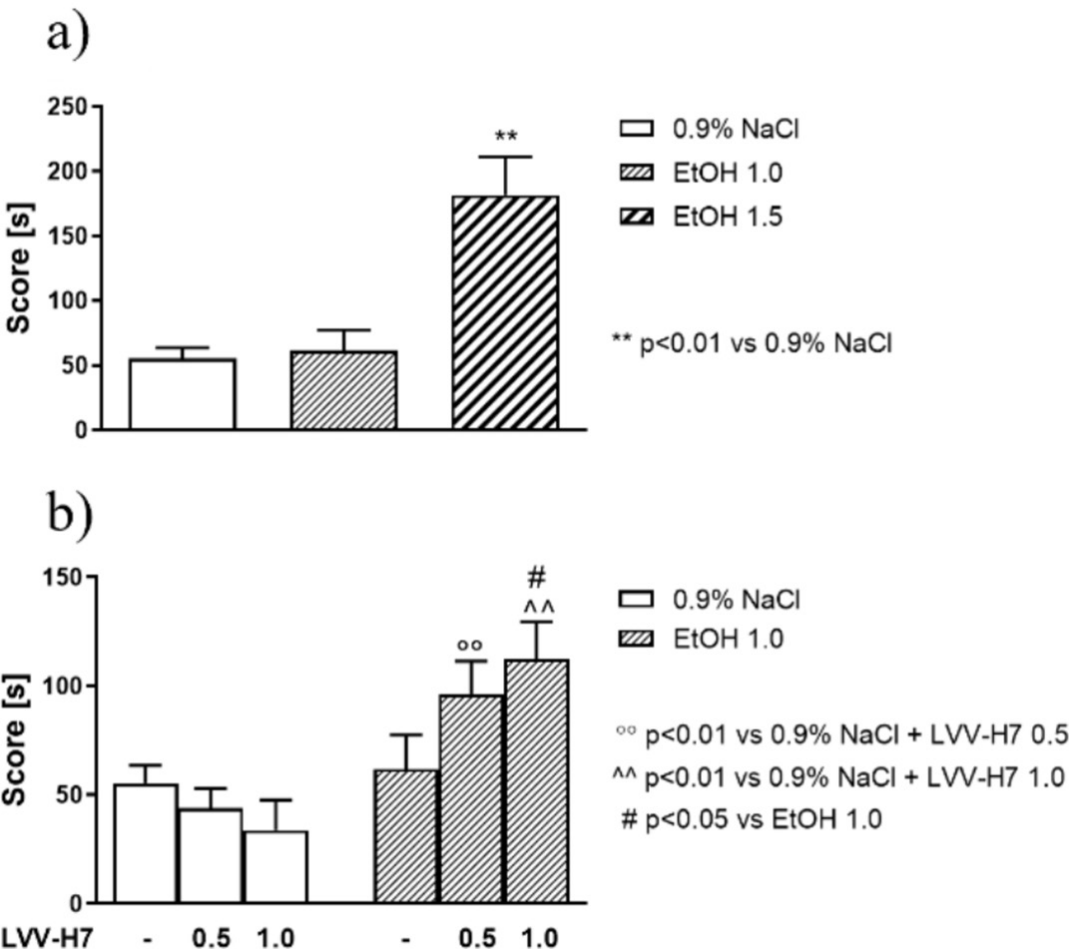
LVV‐H7 effect on ethanol‐CPP test.

The one‐way ANOVA revealed significant differences among the groups [F (2,20) = 11.26, *p* < 0.001] on the CPP test day (Figure 4a). A post hoc analysis (Tukey's test) revealed that the animals spent significantly more time in the drug‐paired compartment on the test day (the expression of ethanol‐induced CPP), following ethanol (1.5 g/kg, i.p.) conditioning, compared with the 0.9% NaCl (control) group (*p* < 0.01). A lower dose of ethanol (1.0 g/kg, i.p.) did not change the time spent by rats in the drug‐associated compartment in the control group (0.9% NaCl) (Figure 4a).

The two‐way ANOVA for the CPP acquisition experiment revealed a significant pretreatment × treatment interaction [F (2,38) = 3474, *p* < 0.01] and an overall effect of pretreatment [F (1,38) = 16.47, *p* < 0.001]. The post hoc analysis (Bonferroni's test) showed that ethanol administered at the dose of 1 g/kg (i.p.) during the acquisition phase did not evoke any rewarding effects on the test day (expression phase) (Figure 4b). However, a single injection of LVV‐H7 before each conditioning session evoked significant ethanol‐CPP at the dose of 1.0 g/kg (*p* < 0.01) (Figure 4b). Moreover, LVV‐H7, given alone at all used doses before each conditioning session, did not change the time spent by rats in the drug‐associated compartment in the control group (0.9% NaCl) (Figure 4b).

### 3.4 Effect of Pepstatin on the Expression of Ethanol‐CPP

**FIGURE 5:**
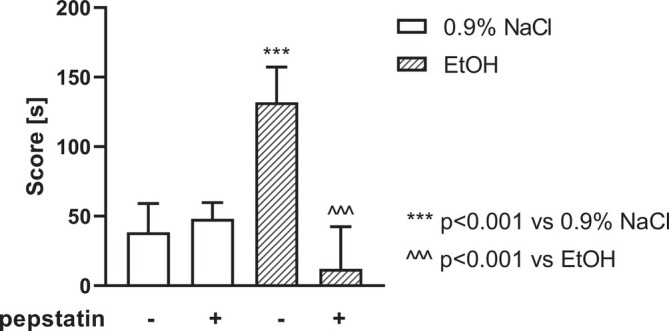
Pepstatin effect on ethanol‐CPP test.

A two‐way ANOVA revealed a statistically significant pretreatment effect [F (1,24) = 10.68, *p* < 0.01], treatment effect [F (1,24) = 39.13, *p* < 0.0001], and a significant pretreatment × treatment interaction [F (2,24) = 53.99, *p* < 0.0001] on the CPP score. The post hoc analysis (Bonferroni's test) revealed that the animals spent more time in the drug‐paired compartment on the test day, following ethanol (1.5 g/kg, 15% w/v) conditioning, as compared with the 0.9% NaCl group (*p* < 0.001). Pepstatin administered before ethanol (1.5 g/kg, 15% w/v, i.p.) at each conditioning session statistically and significantly blocked the expression of ethanol‐CPP (*p* < 0.001). However, pepstatin, given alone at the used dose before each conditioning session, did not change the time spent by rats in the drug‐associated compartment in the control group (0.9% NaCl) (Figure 5).

### 3.5 Studies on Protein Expression in Response to Alcohol Administration

The primary goal of this experiment was to assess potential changes in the expression levels of proteins previously reported in literature to be affected by alcohol administration. These include the μ‐opioid receptor (OPRM1), NMDA receptor, ACE 1 (ACE1), and dopamine cAMP‐regulated neuronal phosphoprotein (DARPP‐32) [26, 27, 28]. To achieve this, we isolated key brain structures involved in alcohol reward, substance seeking, and relapse—specifically, the hippocampus, cortex, striatum, and spinal cords from animals following the CPP test in their respective experimental groups.

**FIGURE 6:**
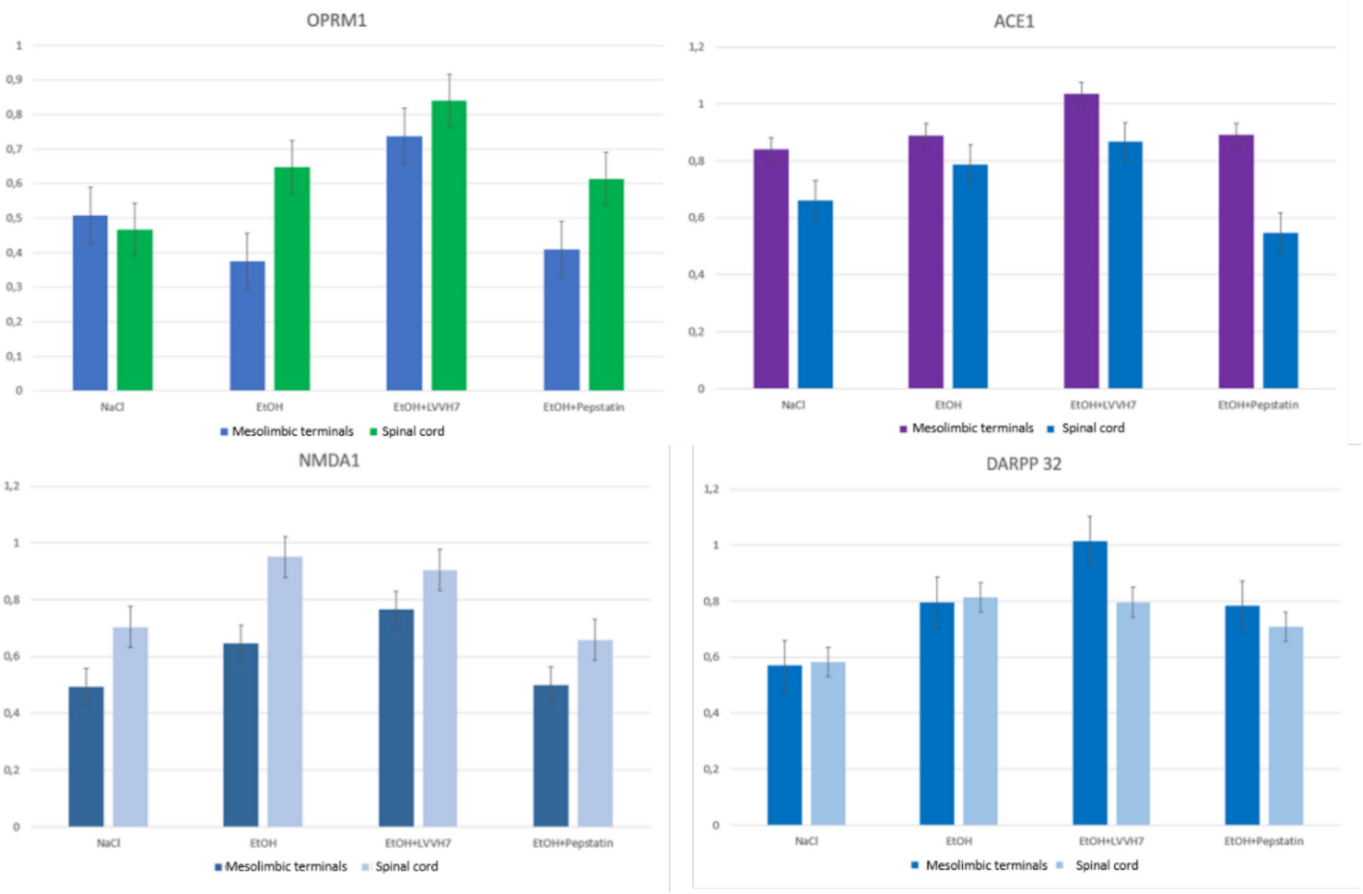
Immunoassay of protein expression levels following EtOH administration and the effect of LVV‐H7 co‐administration.

Protein expression levels were measured using standard Western blotting. SDS‐PAGE was performed after standardizing protein extracts based on total protein concentration (determined via the Bradford method), immunotransfer, and immunodetection. Densitometric analysis was conducted to quantify protein levels in all structures from five animals. The results from brain structures (hippocampus, striatum, and cortex) were pooled and compared according to the alcohol/LVVH7/pepstatin administration (Figure 6).

Our analysis revealed a statistically significant upregulation of NMDA and μ‐opioid receptor (OPRM1) expression following ethanol (EtOH) administration. This effect was even more pronounced in the LVV‐H7 co‐administered group, particularly in the prefrontal cortex and spinal cord, but not in the striatum or hippocampus.

Specifically, OPRM1 expression in the cortex increased from 0.23 ± 0.087 in the control group to 0.43 ± 0.12 in the EtOH/LVV‐H7 co‐administered group. In the spinal cord, OPRM1 levels rose from 0.46 ± 0.1 in controls to 0.84 ± 0.09 in the EtOH/LVV‐H7 group. Interestingly, we confirmed a significant reduction in OPRM1 levels due to alcohol administration in the prefrontal cortex and striatum but not in the hippocampus or spinal cord (detailed data available in Supporting Information). This reduction may result from the development of tolerance to alcohol.

Alcohol is known to inhibit NMDA receptors (NMDAR), and prolonged exposure induces NMDAR upregulation as a compensatory mechanism, contributing to the development of EtOH dependence and withdrawal symptoms. NMDA signalling plays a key role in alcohol‐related behaviours, including reward, intake, seeking and relapses [26]. Our data showed an upregulation of NMDAR following EtOH and LVV‐H7 co‐administration, especially in the spinal cord, where expression increased from 0.7 ± 0.09 in controls to 0.9 ± 0.1 in the EtOH/LVV‐H7 group. We also observed changes in enzyme expression levels, displaying an opposite regulatory trend between brain structures and the spinal cord. While receptor protein levels were higher in the spinal cord, enzyme expression was more pronounced in brain regions.

DARPP‐32 is a key regulator of neurotransmission, acting as an inhibitor of protein phosphatase 1, which is involved in dopaminergic and glutamatergic signalling [29]. As a molecular switch, its activity depends on phosphorylation at specific threonine residues (Thr34/Thr75). Previous studies have shown that DARPP‐32 plays a crucial role in the ethanol reward mechanism, with alcohol increasing Thr34 phosphorylation [30]. DARPP‐32 knockout (KO) mice exhibit significantly reduced ethanol self‐administration [31]. Addiction‐related stimuli have also been shown to trigger DARPP‐32 phosphorylation within the striatum, contributing to behavioural sensitization to psychostimulants such as cocaine and amphetamine. The phosphorylation site shifts from Thr43 to Thr75 during this process. In our study, alcohol administration led to DARPP‐32 upregulation, with even more significant increases observed in the EtOH/LVV‐H7 co‐administered groups across all examined structures. However, the changes were more pronounced in brain regions than in the spinal cord (Supporting Information).

ACE1 inhibition has been shown to effectively reduce alcohol consumption in animals with elevated renin–angiotensin system (RAS) activity, as ACE1 inhibitors also modulate dopamine and corticotropin‐releasing factor levels in the brain [28]. We observed upregulated ACE1 expression in the brain structures of rats co‐administered with EtOH and LVV‐H7. However, significant interindividual variability was detected (Supporting Information), potentially reflecting differences in RAS activity among the studied animals.

### 3.6 Identification of LVV‐H7 Processing Enzymes

**FIGURE 7:**
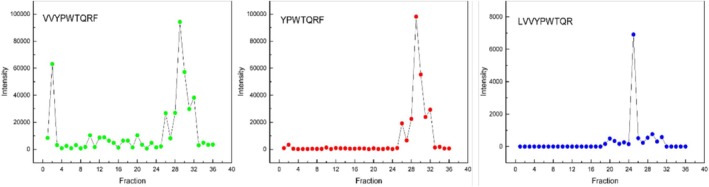
Plots presenting the intensity of LVV‐H7 fragments in homogenate fractions.

Alcohol abuse causes persistent modifications in CNS function, contributing to the development and expression of tolerance, symptoms of withdrawal, and compulsive behaviour focused on obtaining more alcohol. The effects of alcohol involve complex system interactions, and numerous neuropharmacological targets associated with these systems are currently under investigation, including the following: neurotransmission systems (α‐adrenergic, dopaminergic, endocannabinoid, GABAergic, glutamatergic, nicotinic cholinergic, neuropeptide Y, serotonergic and substance P), pathways associated with acetaldehyde‐related enzymes, corticotropin‐releasing factor, feeding‐related peptides, neuroinflammation and nociception. We have developed a proteomic method to identify the sequence of LVV‐H7 degradation enzyme. This method presents some technical limitations and requires robust animal model selection with adequate sample size, accurate sample quality control, sample preparation and/or fractionation, rigorous statistical analysis of results and validation of changes in protein expression using advanced mass spectrometers and bioinformatic tools. Therefore, we have selected spinal cord tissue for this study because this part of the CNS is extensive and presents the best quality parameters for identifying LVV‐H7 processing enzymes. Additionally, the spinal cord's functional properties, while a fundamental building block of ascending and descending addition pathways, transmit messages to the brain from the whole body. Initially, the incubation of the LVV‐H7 with homogenates allowed us to identify various degradation products. This study seems particularly interesting because the biological activity of hemorphins depends on the peptide sequence [32]. Under our experimental conditions, VVYPWTQRF, YPWTQRF, PWTQRF and LVVYPWTQR fragments were mainly revealed. In the case of PWTQRF, the lack of its N‐terminal sequence YPW that determines the opioid activity of hemorphins allowed us to omit this fragment in further consideration. The results obtained are presented in the graphs (Figure 7).

**FIGURE 8:**
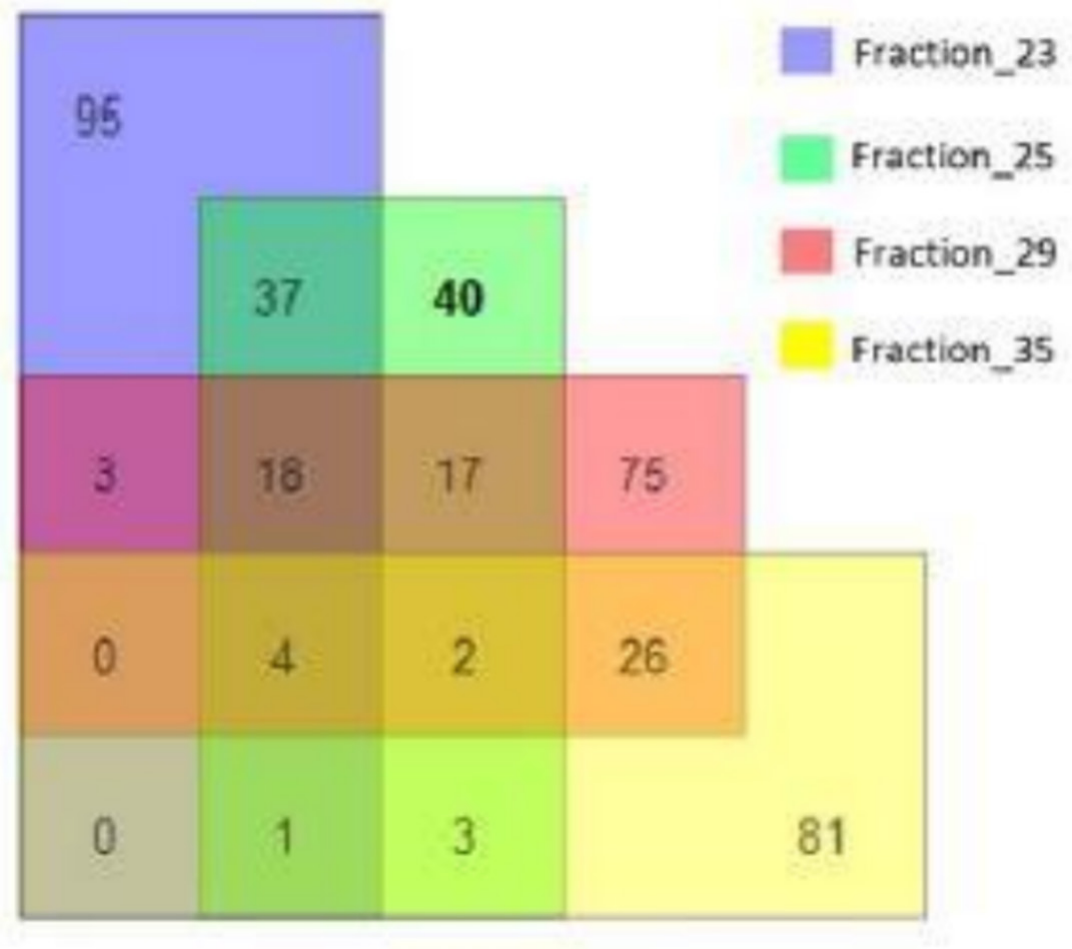
The scheme represents the number of identified proteins.

Due to the obtained data, Fractions 29 (and 35 as a control) and 25 (and 23 as a control) were further concentrated and analysed according to the earlier procedure. It was possible to identify (470) proteins in all samples (Figure 8).

**FIGURE 9:**
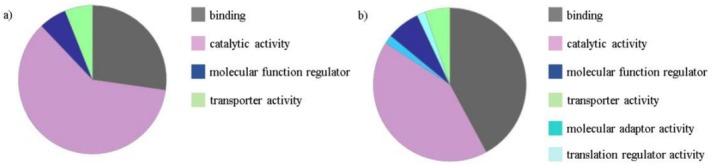
Chart displaying the functional characteristics of the identified proteins: (a) in Fraction 25 and (b) in Fraction 29.

Forty proteins were prevalent in Fraction 25, and 75 were abundant in Fraction 29, absent in the control samples. The proteins were analysed using the Panther Database. The functional characteristics of these proteins are presented in Figure 9.

Based on the biological function, we targeted the LVV‐H7 degrading enzyme among proteins identified in Fractions 25 and 29 (Panther Database). The group with catalytic activity was considered. According to the results, it was impossible to precisely indicate the enzymes implicated in the metabolism of LVV‐H7 concerning the other products. We could select the ones with proteolytic activity from identified enzymes and discuss their potential role in hemorphins' metabolism.

Analysis of Fraction 29 allows the type of puromycin‐sensitive aminopeptidase (PSA) as the enzyme involved in the production of fragment VV‐H7. As the PSA is a zinc‐metallopeptidase that hydrolyzes its substrate's N‐terminal amino acids, it may degrade LVV‐H7 to VV‐H7. It is known that peptide cleavage after digestion by one of the proteases may become substrates for other proteases. Therefore, in the case of the H7 fragment, its generation may occur due to serial aminopeptidases' activity or due to PSA and dipeptidyl peptidases action, which we also identified in the probes. According to Figure 7, we may further observe the presence of the LVV‐H6 fragment. It can, therefore, be assumed that enzymes with carboxypeptidase‐like activity are implicated in hemorphin catabolism. Interestingly, as suggested in Zhao Q.'s work, except for the necessary sequence Tyr‐Pro‐Trp, the phenylalanine in Position 7 of the chain could play a significant role in the opioid potency of hemorphins [33].

### 3.7 Verification of LVV‐H7 Synthetic Peptide Availability on Resin for Enzymatic Cleavage

**FIGURE 10:**
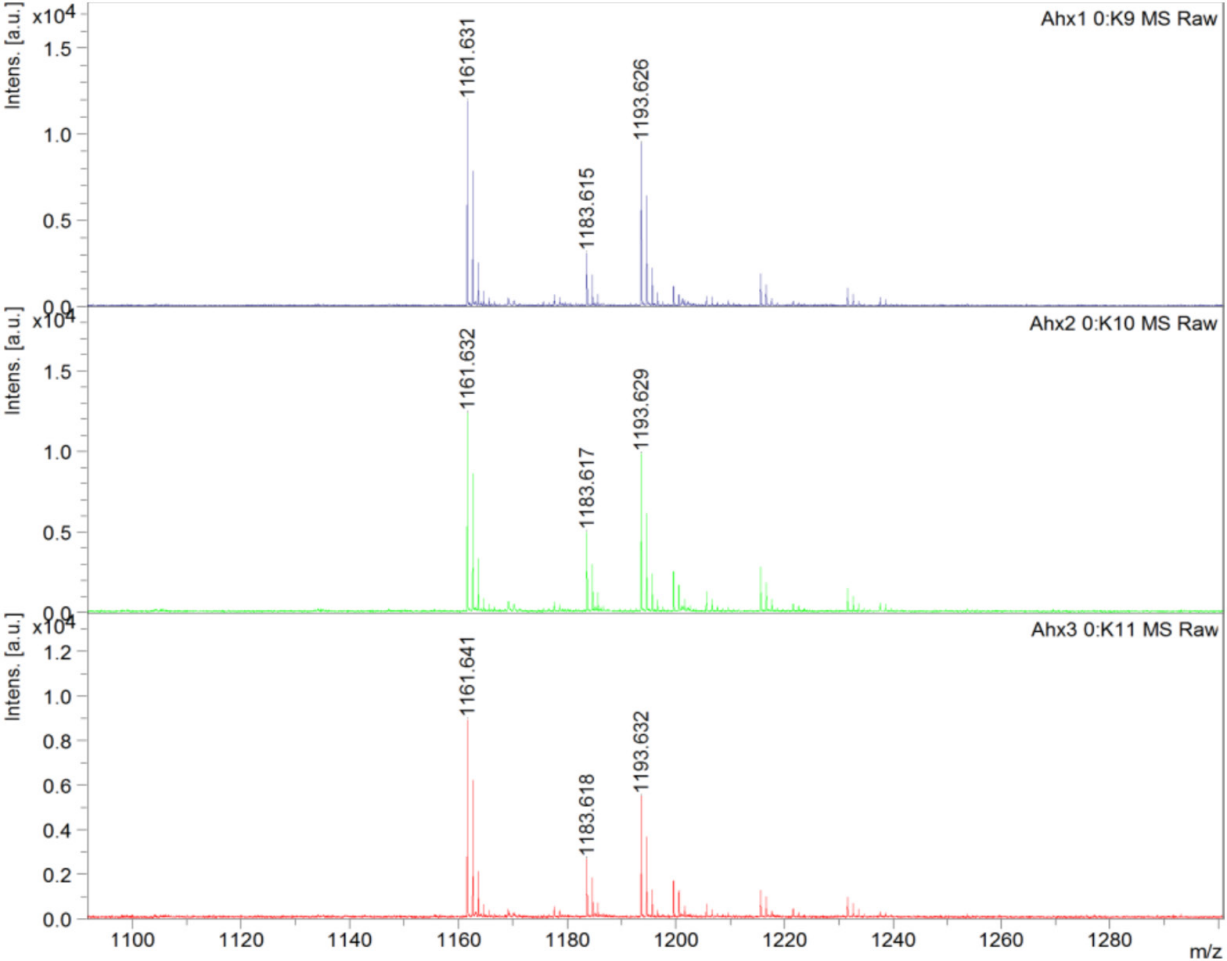
The mass spectra recorded for the mixture were obtained from incubating three synthesized peptidyl‐resins with trypsin solution. From the top, spectra show the result for a peptidyl resin containing one, two and three Ahx linkers, respectively.

Mass spectra recorded for the LVV‐H7 digests obtained because of incubation of three synthesized peptidyl‐resins with trypsin solution are presented in Figure 10. From the top, spectra show the result for a peptidyl‐resin containing one, two and three Ahx linker residues, respectively, creating different lengths of the total linker. The peak at 1161.6 m/z corresponds to the MH^+^ ion for the cleaved peptide (Leu‐Val‐Val‐Tyr‐Pro‐Trp‐Thr‐Gln‐Arg). The peaks at 1183.6 and 1193.632 m/z correspond to sodium and potassium adducts of the tested peptide.

Based on the obtained results, it can be concluded that the amount of Ahx acid linkers does not significantly affect steric hindrance and efficiency of enzymatic digestion of LVV‐hemorphin immobilized on the TentaGel resin.

The fragment mass spectra confirmed the amino acid sequence of the enzyme product of peptidyl‐resin digestion. An exemplary fragmentation spectrum of the cleaved Leu‐Val‐Val‐Tyr‐Pro‐Trp‐Thr‐Gln‐Arg is shown in Figure 2. The correct sequence of the obtained peptidyl‐resin digestion product by trypsin confirmed the sequence of the LVV‐hemorphin peptide synthesis on the resin. Fragment spectra for the ion 1161.6 m/z allow us to determine the amino acid sequence of the cleaved peptide (Leu‐Val‐Val‐Tyr‐Pro‐Trp‐Thr‐Gln‐Arg). The amino acid sequence was assigned using the BioTools software from Bruker Daltonics.

The synthesis of peptides on a solid support for isolating binding counterparts from the CNS using affinity chromatography was successfully achieved.

### 3.8 Identification of LVV‐H7 Binding Partners in Response to Alcohol Dependence in the Brain and Spinal Cord

Proteins bound to the LVV‐H7 sequence, specific to the brain tissue in both groups of rats treated with ethanol and the control group, were identified by liquid chromatography combined with mass spectrometry. Table S1 presents a list of all identified proteins.

**FIGURE 11:**
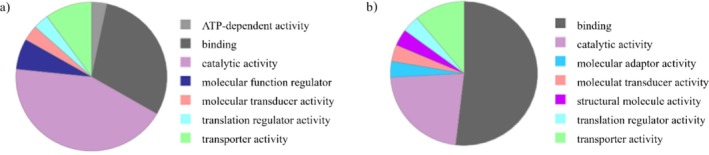
Functional‐GO analysis by Panther Database of the regulated proteins binding to the LVV‐H7 sequence characteristic of the brain tissue in both groups of rats: (a) the control group and (b) treated with ethanol.

Alpha‐synuclein (SNCA), glycogen synthase kinase‐3 beta (GSK3B), fatty acid synthase (FASN) and ATP synthase subunit beta, mitochondrial (ATP5B) were identified. All play essential roles in releasing neurotransmitters into the synaptic cleft and stabilizing the formation of biphasic systems. Results of the functional‐GO analysis of the identified proteins carried out by the Panther Database are collected in Figure 11. Functional GO‐analysis shows that catalytic activity is the primary molecular function for the proteins identified only in the control group, while binding molecular function is the most pronounced for proteins identified only in the alcohol‐treated group (Figure 11). Also, no regulatory role is exerted on molecular function in the alcohol‐treated group. Simultaneously, proteins with molecule activity occur.

Among proteins interacting with the LVV‐H7 sequence, SNCA was identified in control and alcohol‐treated groups (each group was analysed in biological triplicates). Also, several other proteins exhibit a high number of interactions, such as GSK3B, stress‐70 protein, mitochondrial (HSPA9), heat shock protein HSP 90‐beta (HSP90AB1), T‐complex protein 1 subunit alpha (TCP1), ATP synthase subunit beta, mitochondrial (ATP5B) and elongation factor 1‐alpha 1 (EEF1A1).

**FIGURE 12:**
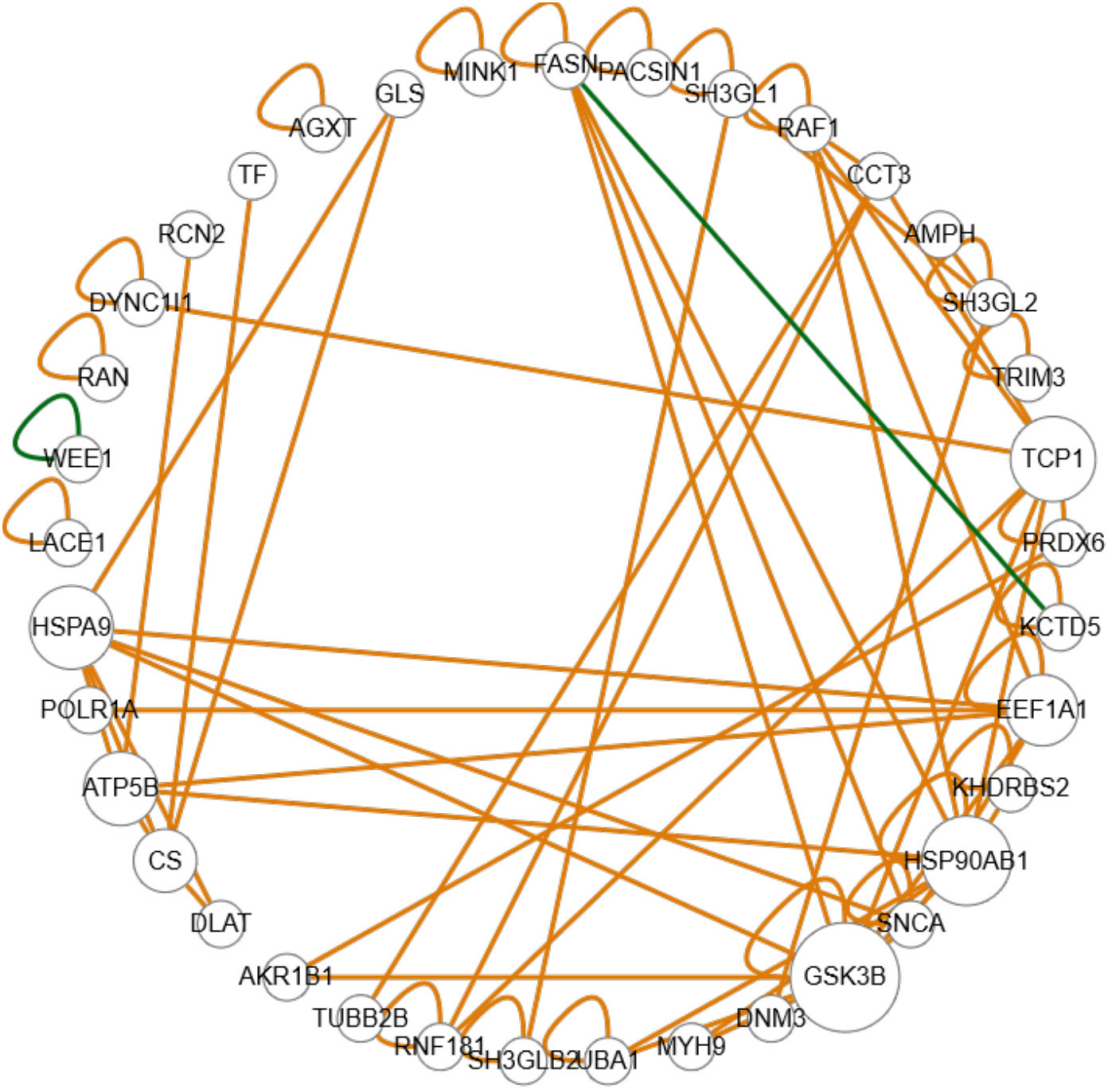
Genetic (green) and protein (brown) interaction networks between regulated proteins' binding to the LVV‐H7 sequence characteristic of the brain tissue in both groups of rats treated with ethanol and the control group. Interactions are established by the easy from the bioGRID database (human interactions).

SNCA also has physical interactions with GSK3B and EEF1A1 genes, and some other interactions are present with HSPA9 and FASN. The size of the nodes represents which nodes are the most central in the network (Figure 12). We treat the network as made of directed edges and calculate the betweenness centrality for each node, equal to the shortest paths that pass through that node. Also, two genetic interactions are present. One is between fatty acid synthase (FASN) and BTB/POZ domain‐containing protein KCTD5 (KCTD5).

The SNCA represents a few main interactions. However, there are interesting interactions with some other proteins. The first is with T‐complex protein 1 subunit gamma (CCT3), a chaperonin‐containing T‐complex component. This molecular chaperone complex assists protein folding upon ATP hydrolysis [34]. The second is RAF proto‐oncogene serine/threonine‐protein kinase (RAF1), which is a kinase that acts as a regulatory link between the membrane‐associated Ras GTPases and the MAPK/ERK cascade, and this regulatory pathway comprises critical cell fate decisions, including proliferation, apoptosis, differentiation, survival, and oncogenic transformation [35]. Elongation factor 1‐alpha 1 (EEF1A1) is a protein that promotes the GTP‐dependent binding of aminoacyl‐tRNA to the A‐site of ribosomes, which occurs during protein biosynthesis [34]. The following interaction includes heat shock protein HSP 90‐beta (HSP90AB1), which promotes maturation and proper regulation of specific target proteins such as cell cycle control and signal transduction. It is the second protein with the highest number of identified interactions. This protein also promotes cell differentiation and, as a result, protects from auto‐ubiquitination and degradation by the proteasomal machinery [34]. The final interaction of the SNCA with GSK3B, a protein kinase that acts as a negative regulator in the hormonal control of glucose homeostasis. This protein also has an anti‐apoptotic function, which is most effective with weak apoptotic signals but can be overcome by stronger stimulation [34] [36]. Besides this, GSK3B represents the highest number of interactions from all identified proteins, along with SNCA. The stimulatory effect of SNCA on the tau‐phosphorylation by GSK3B was reported [37]. Networks representing interactions of identified genes with all other genes are presented in Figure S6.

## 4 Discussion

Addiction is a pathological form of neuroplasticity, along with the emergence of aberrant behaviours involving a cascade of neurochemical changes, mainly in the brain's reward system. Understanding the molecular mechanisms of behavioural sensitization would facilitate the discovery of drug therapy programmes against alcohol addiction. The concentration of hemorphins and their cleavage under physiological and pathophysiological conditions are not fully understood. Hemorphins act as therapeutic reagents (addiction, pain treatment, memory deficits, cancer, inflammation, anticonvulsants, blood pressure control, vascular and renal system control, Alzheimer's disease, antidepressants and biomarkers) in various diseases.

Hemorphins, through their interaction with MORs, can modulate the response in AUD. Studies suggest that hemorphins may either potentiate or inhibit opioid receptor activity, depending on their concentration and receptor affinity. In the context of AUD, elevated levels of hemorphins might enhance opioid signalling, thereby reinforcing alcohol‐seeking behaviour. Conversely, changes in hemorphin activity could contribute to withdrawal symptoms by disrupting opioid homeostasis.

When hemorphins enhance MOR signalling, dopamine release in the brain is elevated, reinforcing alcohol consumption. On the other hand, changes in hemorphin levels during withdrawal might lead to decreased dopamine release, contributing to negative affective states and increasing the likelihood of relapse.

Similarly, alcohol disrupts glutamatergic signalling by inhibiting NMDA receptors. Chronic alcohol use results in NMDA receptor upregulation, contributing to withdrawal excitotoxicity. Hemorphins may indirectly interact with glutamatergic pathways, possibly affecting NMDA receptor activity through opioid system modulation. Understanding these interactions could provide insights into new therapeutic targets for managing withdrawal symptoms.

Modulating hemorphin activity could help restore neurotransmitter balance, alleviate withdrawal symptoms, and reduce alcohol cravings. Pharmacological approaches that influence hemorphin levels or their receptor interactions could be explored. For instance, inhibitors of hemorphin‐degrading enzymes might prolong hemorphin activity (including PSA), providing sustained opioid receptor modulation. Additionally, synthetic hemorphin analogues could be developed to target specific receptor subtypes involved in alcohol dependence selectively.

## 5 Conclusion

Hemorphins play a multifaceted role in alcohol dependence by modulating opioid, dopaminergic, and NMDA pain‐related pathways. Their interactions with the MOR and other neurotransmitter systems contribute to the reinforcing effects of alcohol, withdrawal symptoms and stress responses. Understanding these mechanisms could pave the way for novel therapeutic strategies in AUD treatment.

In this study, we investigated the in vivo role of the cryptide LVV‐H7 in alcohol‐related reward using a combination of proteomic and behavioural approaches. Synthetic LVV‐H7 was administered to rats in a CPP paradigm, with animals receiving intraperitoneal injections of alcohol, LVV‐H7, pepstatin (a cathepsin D inhibitor that prevents LVV‐H7cleavage), NaCl or combinations thereof.

We assessed protein expression changes in brain regions associated with alcohol‐related reward, focusing on the MOR, NMDA receptor, DARPP‐32 and ACE. Immunoassays revealed significant upregulation of NMDA and MOR expression in the prefrontal cortex and spinal cord, particularly in the ethanol/LVV‐H7 co‐administered group. Enzymatic activity of LVV‐H7‐degrading enzymes was quantified using cathepsin D‐specific substrates, both in vivo and in vitro, with spinal cord tissue identified as a key site for LVV‐H7 metabolism. The synthesis of LVV‐H7 was optimized on solid‐phase support using standard Fmoc strategies, ensuring efficient enzymatic digestion and structural integrity. Vascular permeability studies demonstrated that both LVV‐H7 and pepstatin successfully crossed the BBB, as confirmed by fluorescence microscopy.

Behavioural analysis revealed that LVV‐H7 significantly enhanced ethanol‐induced CPP at a lower ethanol dose (1.0 g/kg), suggesting a potentiating effect. At the same time, pepstatin administration blocked the expression of ethanol‐CPP, implicating LVV‐H7 cleavage in the rewarding properties of alcohol.

Proteomic identification of LVV‐H7 degradation products revealed multiple bioactive fragments, with PSA implicated in producing VV‐H7 through N‐terminal cleavage. Further mass spectrometry analysis of LVV‐H7 binding partners highlighted key interactions with SNCA, GSK3B and ATP synthase subunit beta (ATP5B). Functional GO analysis indicated that binding molecular functions were prominent in the alcohol‐treated group, suggesting a shift in protein interaction dynamics.

Our findings support the hypothesis that LVV‐H7 plays a significant role in modulating alcohol reward and dependence mechanisms, acting through specific protein–protein interactions and enzymatic pathways. This study offers new insights into the molecular underpinnings of alcohol addiction and identifies LVV‐H7 as a potential therapeutic target.

## Author Contributions

Conceptualization: a.d., E.Y‐K.H., J.H.K. and J.S. Methodology: P.M., K.H., a.d., T.S. and J.H.K. Validation: P.M. and a.d. Formal analysis: P.M., K.H., a.d. and J.H.K. Investigation: P.M., a.d., E.G‐T., P.G., T.S. and J.H.K. Writing – original draft preparation: P.M., K.H., a.d. and J.H.K. Writing – review and editing: E.Y‐K.H. and J.S. Visualization: a.d. Supervision: J.S. Project administration: P.M. and a.d. Funding acquisition: a.d., P.M. and J.S. All authors have read and agreed to the published version of the manuscript.

## Ethics Statement

The study was conducted according to the guidelines of the Declaration of Helsinki and the National Institute of Health Guidelines for the Care and Use of Laboratory Animals, as well as to the European Community Council Directive for Care and Use of Laboratory Animals (86/609/EEC) and was approved by the Local Ethics Committee. Additionally, we confirm that the study is reported following ARRIVE guidelines.

## Conflicts of Interest

The authors declare no conflicts of interest.

## Supporting information


**Figure S1:** Immunoassay of OPRM1 expression level as a result of EtOH administration and the effect of LVV‐H7 co‐administration.
**Figure S2:** Immunoassay of ACE1 expression level as a result of EtOH administration and the effect of LVV‐H7 co‐administration.
**Figure S3:** Immunoassay of NMDA1 expression level as a result of EtOH administration and the effect of LVV‐H7 co‐administration.
**Figure S4:** Immunoassay of DARPP32 expression level as a result of EtOH administration and the effect of LVV‐H7 co‐administration.
**Figure S5:** Exemplary scans of blots (a) ACE1, (b) DARP32, (c) NMDA1, and (d) OPRM1 in studied animal groups.
**Table S1:** Identified proteins bound to the LVV‐H7 sequence, specific to the brain tissue in both group of rats treated with ethanol (Meta Score A) and the control group (Meta Score B).
**Figure S6:** Genetic (green) and physical (brown) interaction networks between regulated proteins' binding to the LVV‐H7 sequence characteristic of the brain tissue in both group of rats treated with ethanol and the control group with all other genes in the database. Interactions established by the easyN from bioGRID database (human interactions).

## Data Availability

The mass spectrometry proteomics data have been deposited to the ProteomeXchange Consortium [38] (https://www.proteomexchange.org) via the PRIDE partner repository [39] with the dataset identifier PXD036448. This published article and its Supporting Information files include the western blotting data generated during this study.
