# Supplementary material for: The Role of LVV‐H7 in Alcohol‐Induced Reward Mechanisms
Source: Addict Biol. 2025 Sep 5;30(9):e70086. doi: 10.1111/adb.70086 (PMC12412044; doi:10.1111/adb.70086)
Supplement: Supplementary file 1 — Figure S1: Immunoassay of OPRM1 expression level as a result of EtOH administration and the effect of LVV‐H7 co‐administration. Figure S2: Immunoassay of ACE1 expression level as a result of EtOH administration and the effect of LVV‐H7 co‐administration. Figure S3: Immunoassay of NMDA1 expression level as a result of EtOH administration and the effect of LVV‐H7 co‐administration. Figure S4: Immunoassay of DARPP32 expression level as a result of EtOH administration and the effect of LVV‐H7 co‐administration. Figure S5: Exemplary scans of blots (a) ACE1, (b) DARP32, (c) NMDA1, and (d) OPRM1 in studied animal groups. Table S1: Identified proteins bound to the LVV‐H7 sequence, specific to the brain tissue in both group of rats treated with ethanol (Meta Score A) and the control group (Meta Score B). Figure S6: Genetic (green) and physical (brown) interaction networks between regulated proteins' binding to the LVV‐H7 sequence characteristic of the brain tissue in both group of rats treated with ethanol and the control group with all other genes in the database. Interactions established by the easyN from bioGRID database (human interactions). [file ADB-30-e70086-s001.pdf]

Supplementary materials

The role of LVV-H7 in alcohol dependence

Przemyslaw Mielczarek [1,2*], Kinga Hartman [1], Eagle Yi-Kung Huang [3,4], Ewa Gibula-Tarlowska [5], Pawel Grochecki [5], Tymoteusz Slowik [6], Jolanta H. Kotlinska [5], Jerzy Silberring [1] and Anna Drabik [1]

[1] Faculty of Materials Science and Ceramics, AGH University of Krakow, Mickiewicza Ave. 30, 30-059 Krakow, Poland
[2] Laboratory of Proteomics and Mass Spectrometry, Maj Institute of Pharmacology, Polish Academy of Sciences, Smetna 12, 31-343 Krakow, Poland
[3] Department of Pharmacology, National Defense Medical Center, Taipei 11490, Taiwan
[4] Graduate Institute of Medical Sciences, National Defense Medical Center, Taipei 11490, Taiwan
[5] Department of Pharmacology and Pharmacodynamics, Faculty of Pharmacy With Division of Medical Analytics, Medical University of Lublin, 4a Chodzki Str., 20-093 Lublin, Poland
[6] Experimental Medicine Center, Medical University of Lublin, Jaczewskiego 8D Str., 20-090 Lublin, Poland
* Correspondence: przemyslaw.mielczarek@agh.edu.pl; Tel.: + 48 12 617 5083

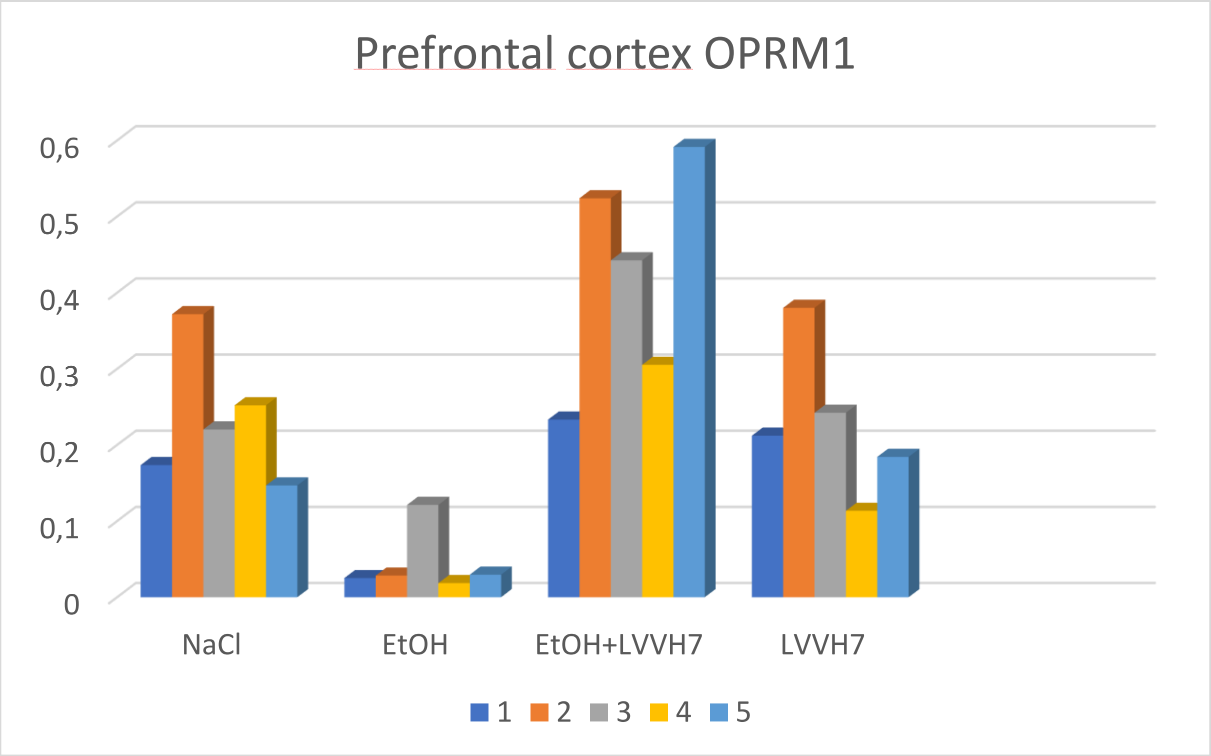

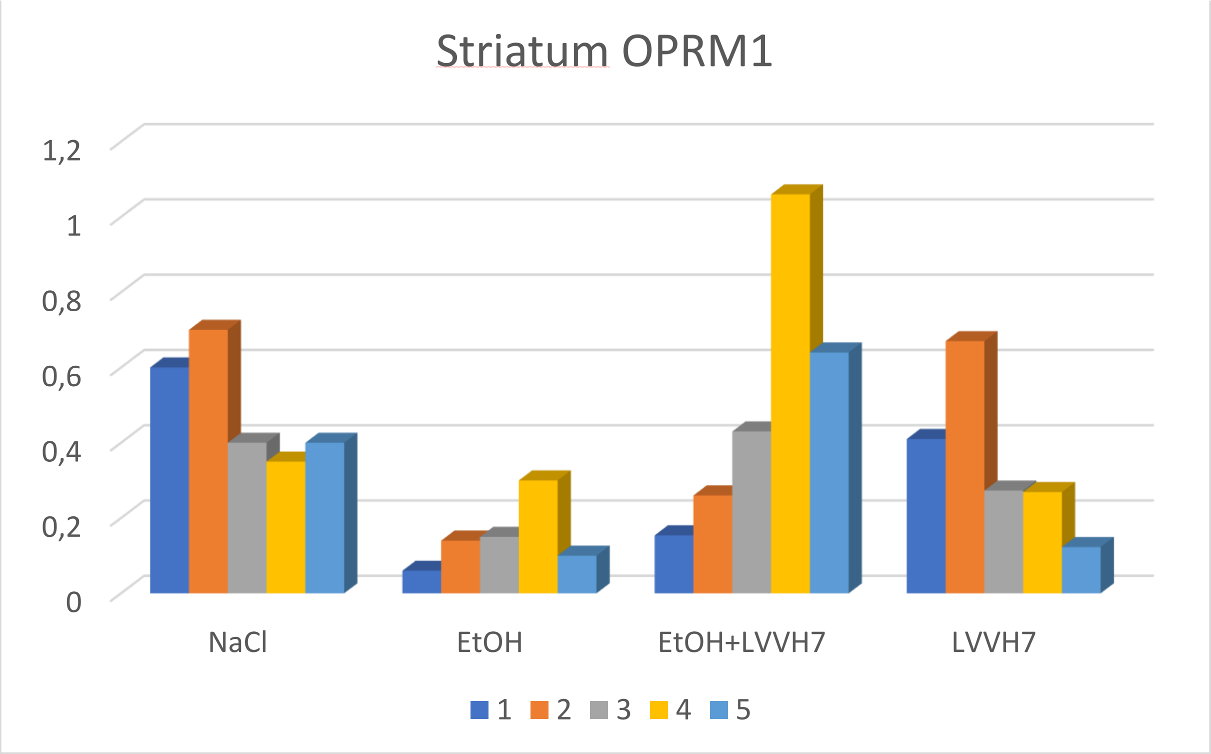
Striatum OPRM1
1,2
1
0,8
0,6
0,4
0,2
0
NaCl
EtOH
EtOH+LVVH7
LVVH7
1 2 3 4 5

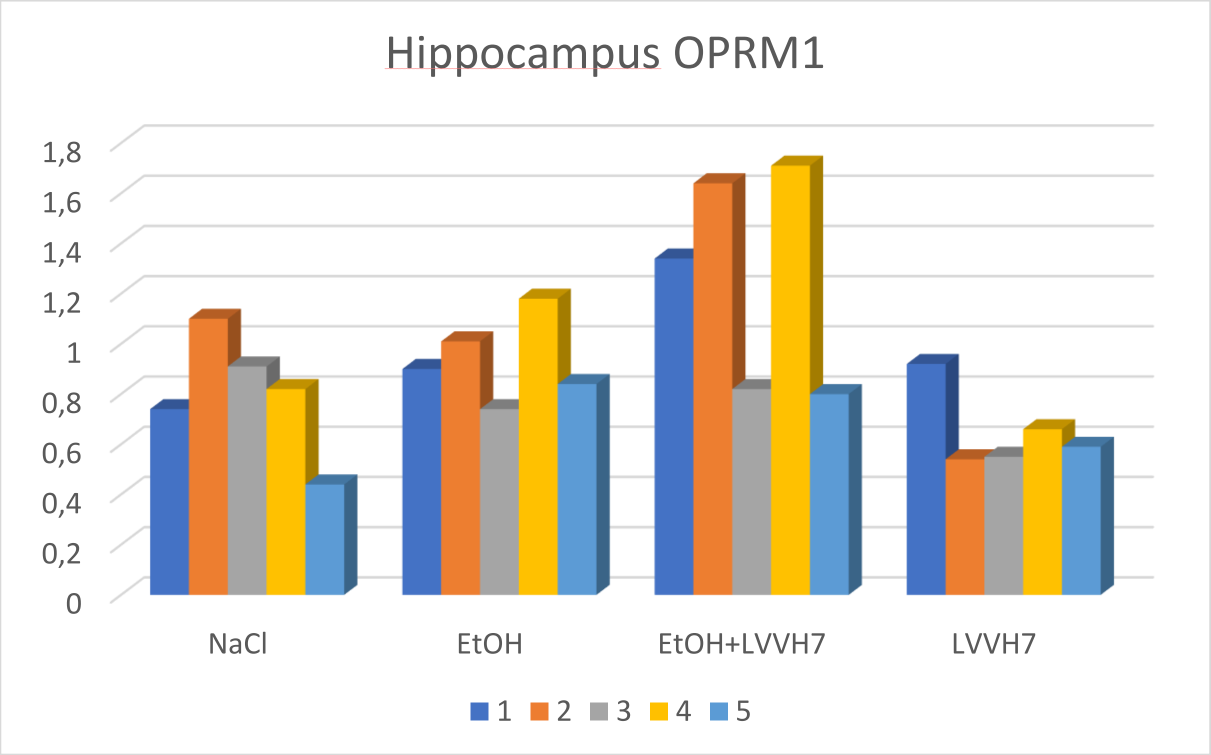
Hippocampus OPRM1
1,8
1,6
1,4
1,2
1
0,8
0,6
0,4
0,2
0
NaCl
EtOH
EtOH+LVVH7
LVVH7
1 2 3 4 5

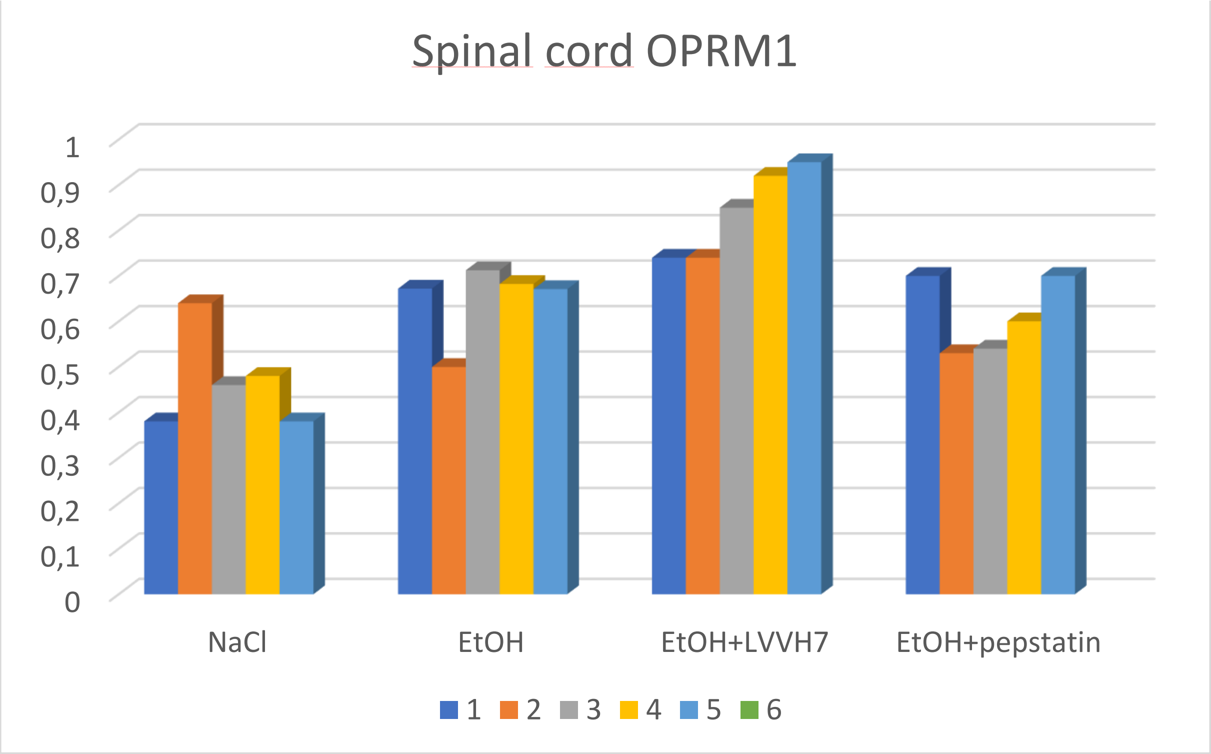

Figure S1. Immunassay of OPRM1 expression level as a result of EtOH administration and the effect of LVV-H7 co-administration.

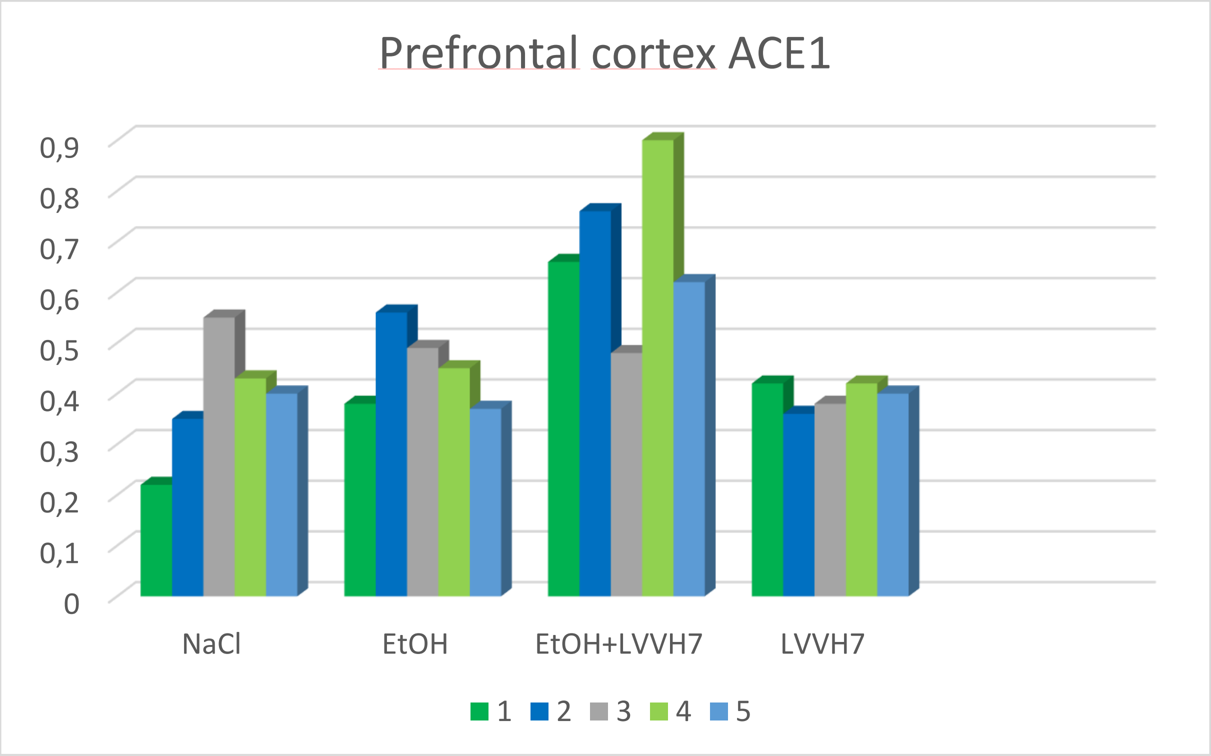

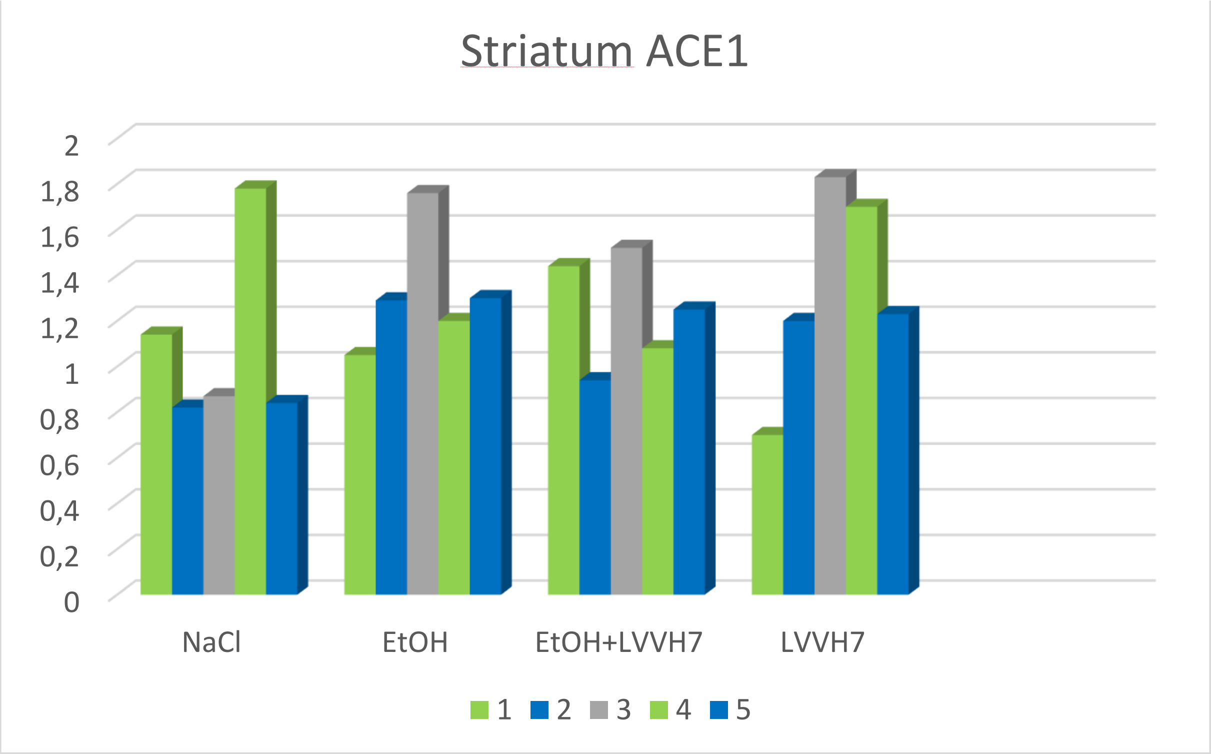
Striatum ACE1
2
1,8
1,6
1,4
1,2
1
0,8
0,6
0,4
0,2
0
NaCl
EtOH
EtOH+LVVH7
LVVH7
1 2 3 4 5

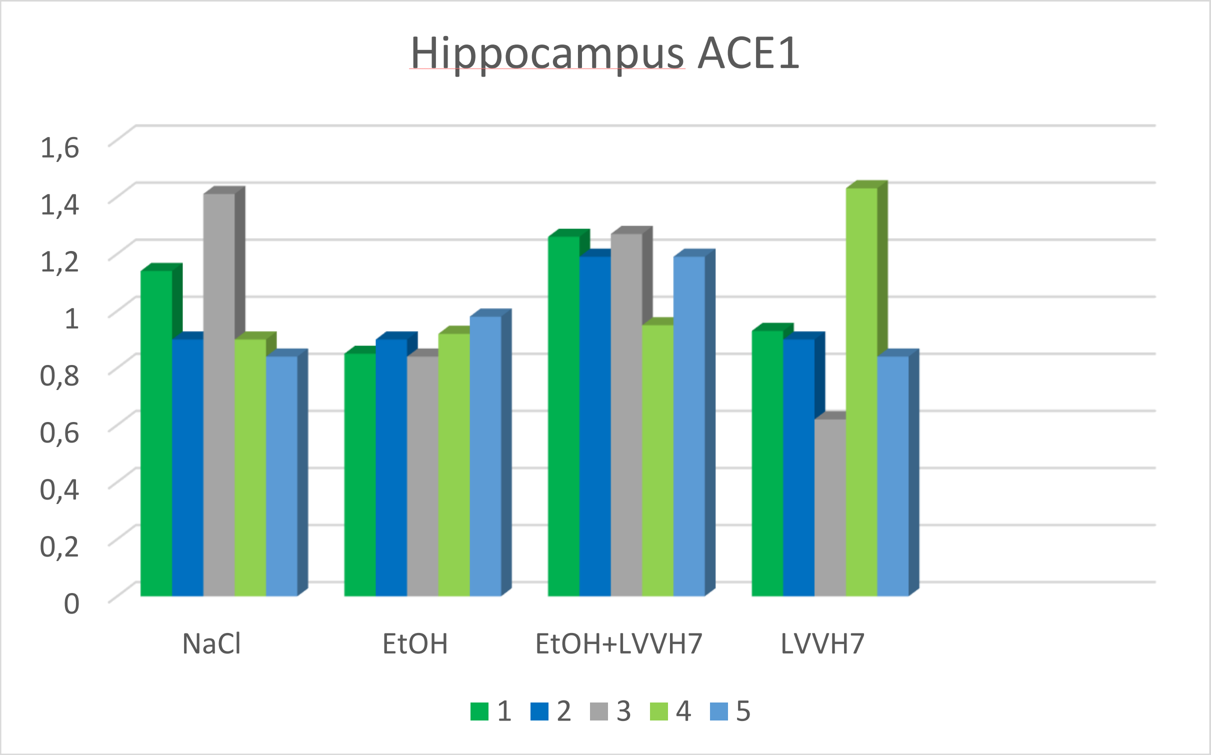
Hippocampus ACE1
1,6
1,4
1,2
1
0,8
0,6
0,4
0,2
0
NaCl
EtOH
EtOH+LVVH7
LVVH7
1 2 3 4 5

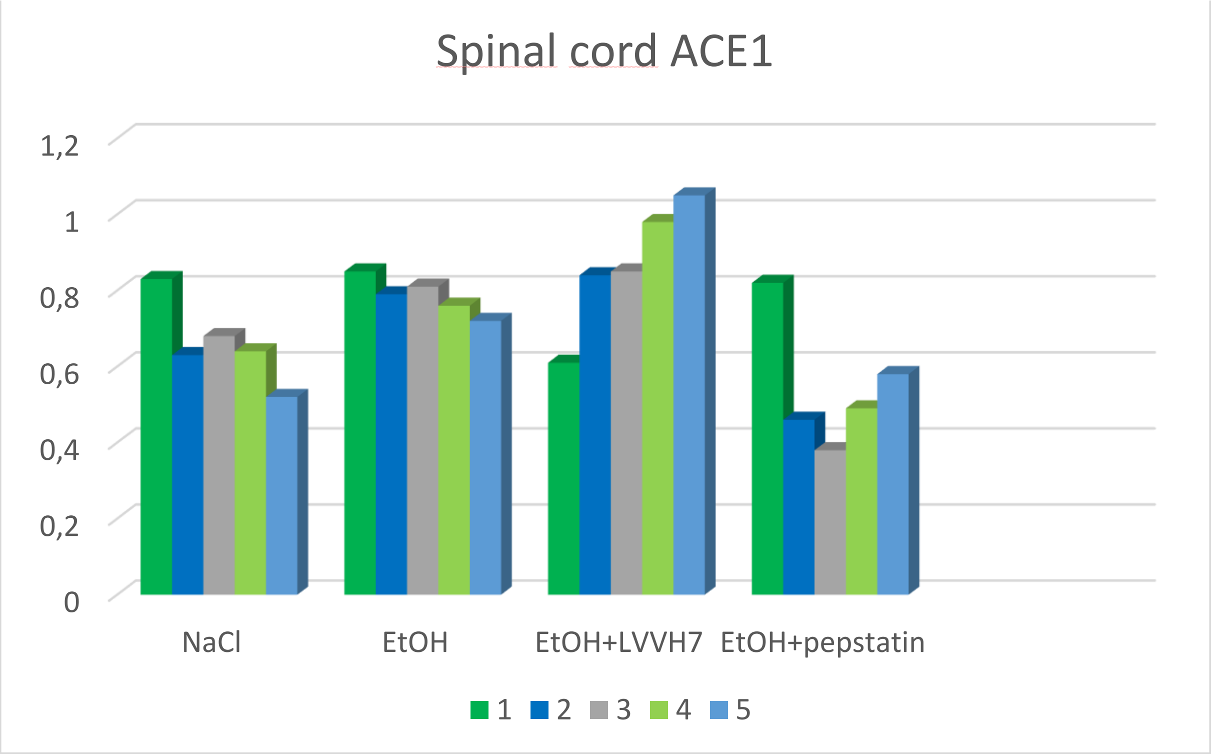

Figure S2. Immunassay of ACE1 expression level as a result of EtOH administration and the effect of LVV-H7 co-administration.

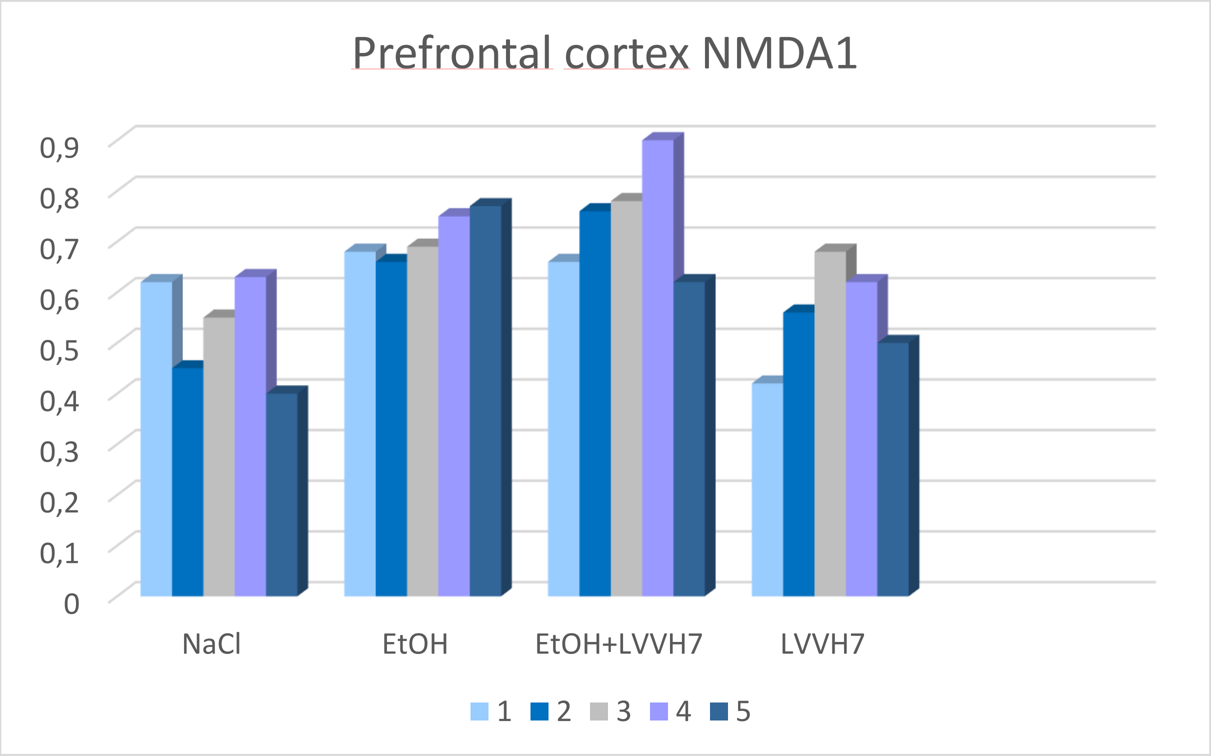

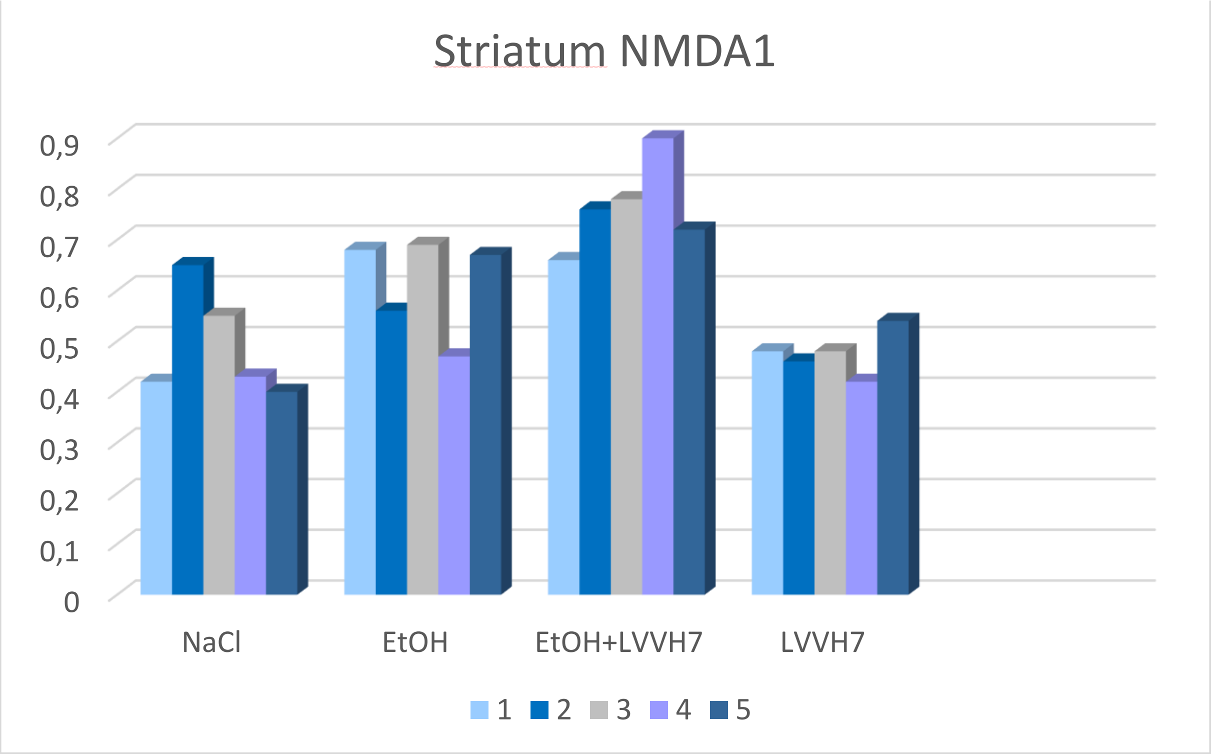
Striatum NMDA1
0,9
0,8
0,7
0,6
0,5
0,4
0,3
0,2
0,1
0
NaCl
EtOH
EtOH+LVVH7
LVVH7
1 2 3 4 5

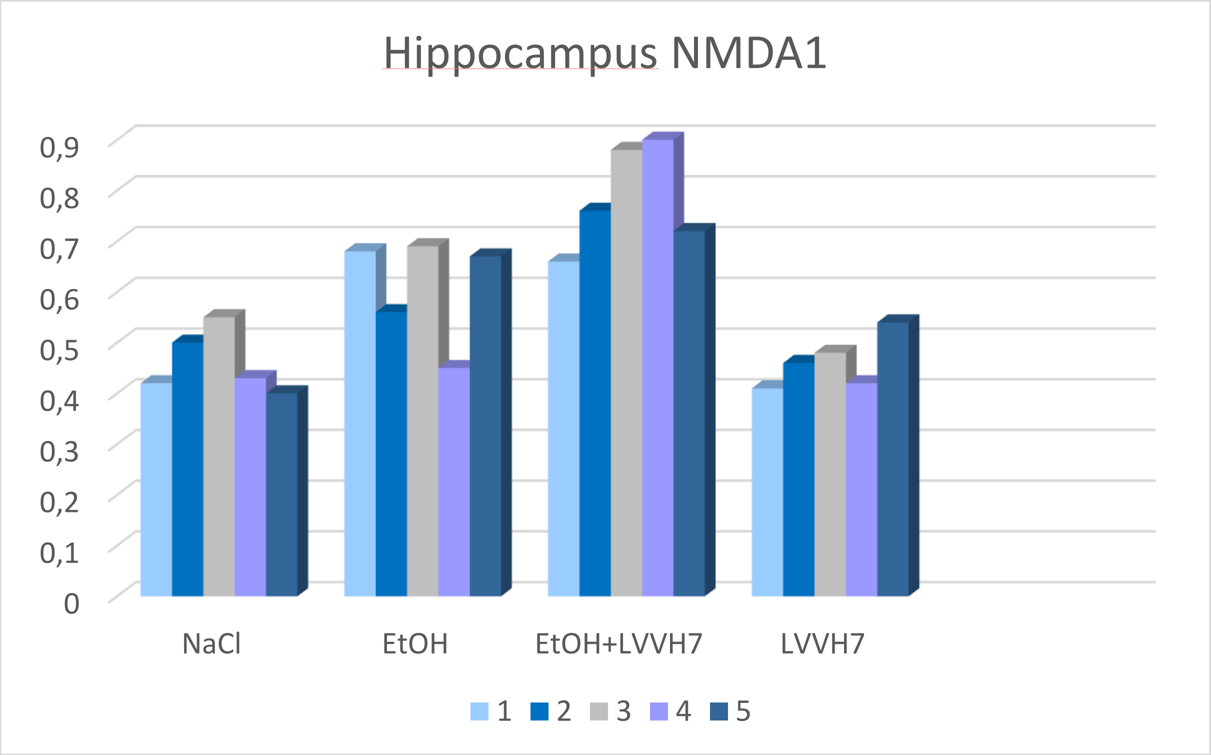
Hippocampus NMDA1
0,9
0,8
0,7
0,6
0,5
0,4
0,3
0,2
0,1
0
NaCl
EtOH
EtOH+LVVH7
LVVH7
1 2 3 4 5

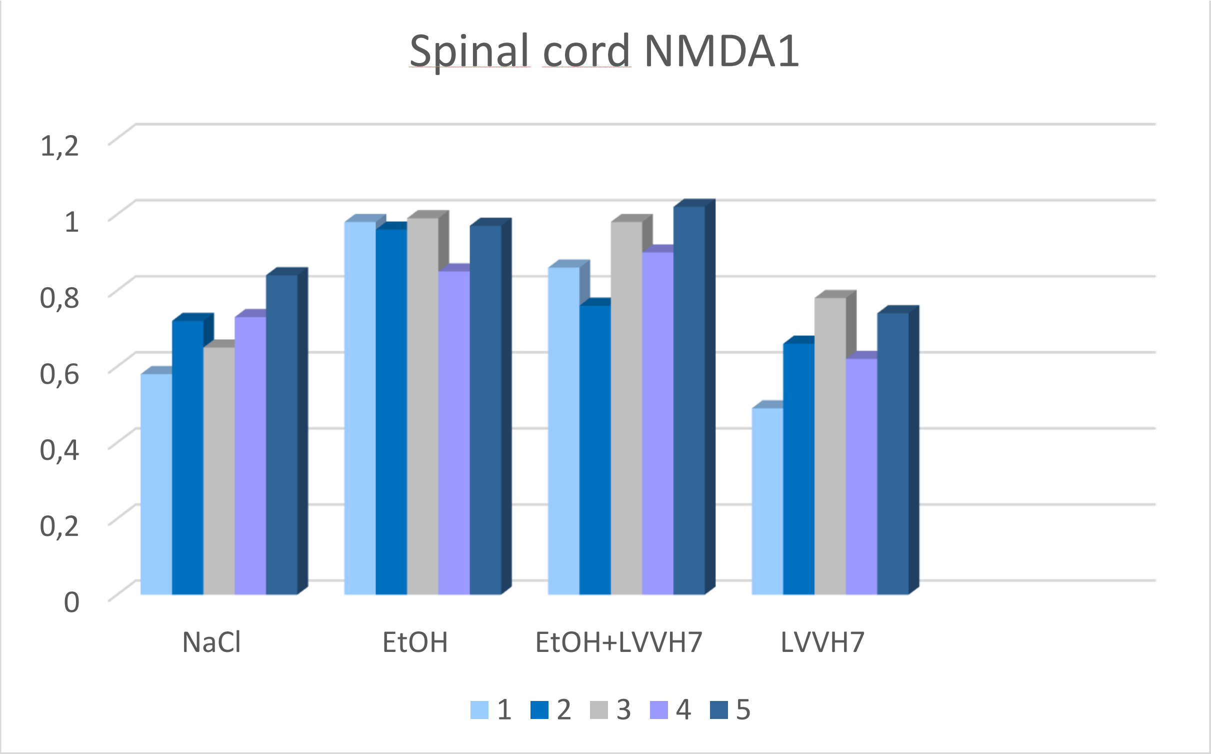

Figure S3. Immunassay of NMDA1 expression level as a result of EtOH administration and the effect of LVV-H7 co-administration.

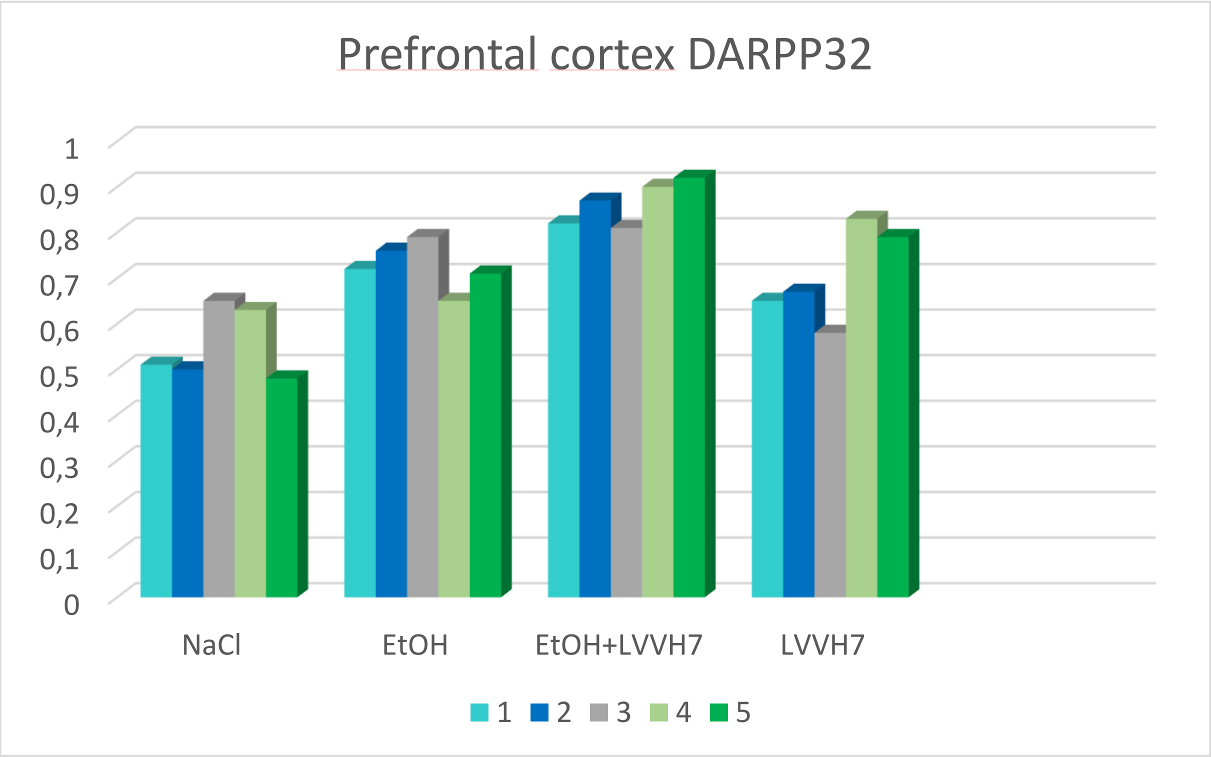

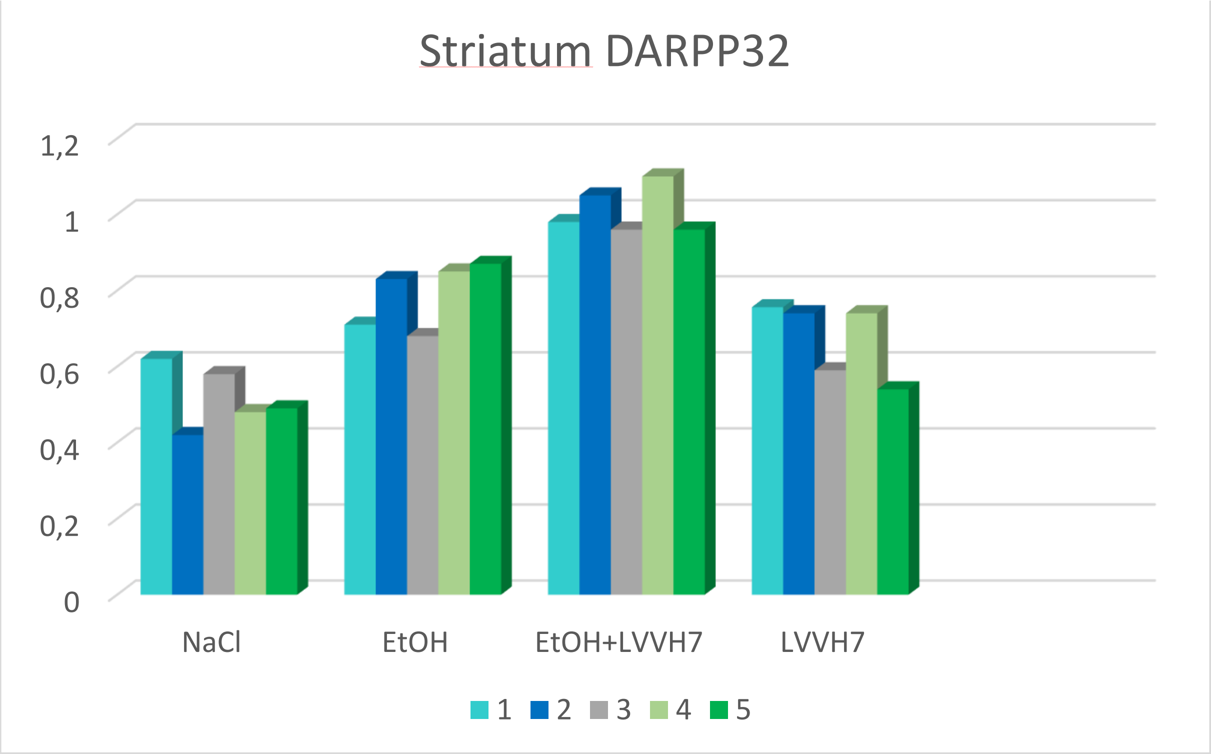
Striatum DARPP32
1,2
1
0,8
0,6
0,4
0,2
0
NaCl
EtOH
EtOH+LVVH7
LVVH7
1
2
3
4
5

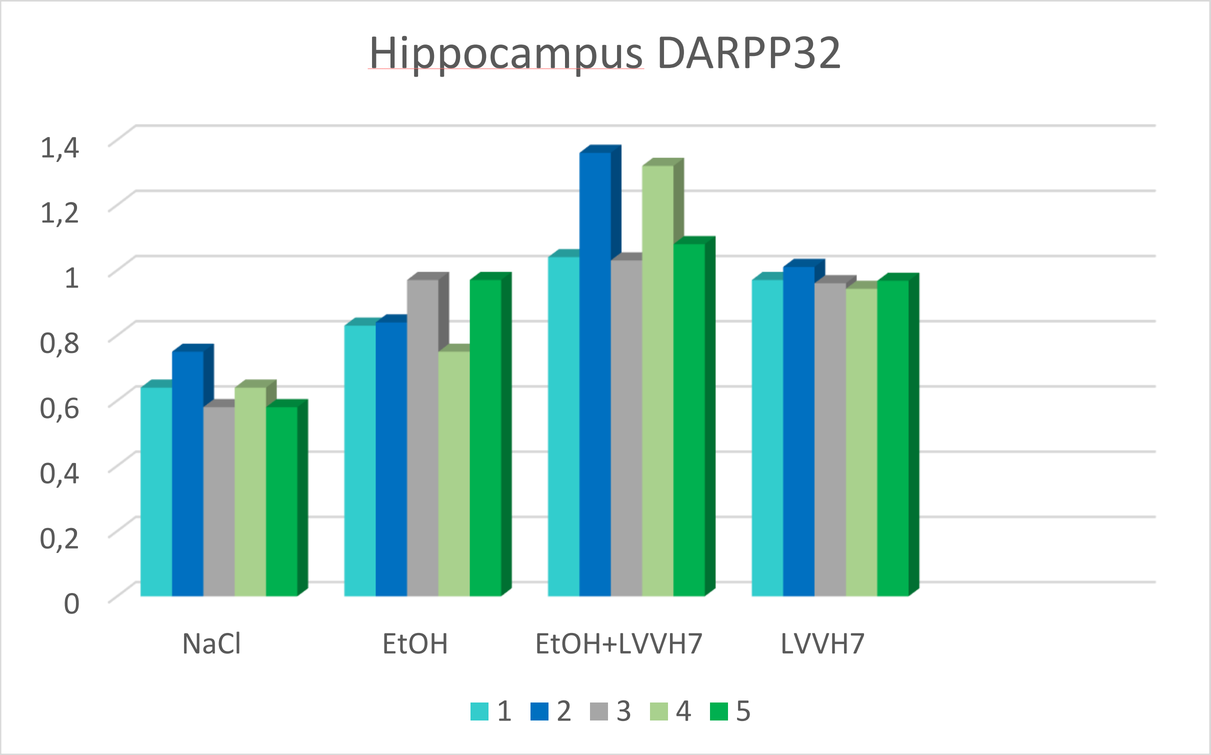
Hippocampus DARPP32
1,4
1,2
1
0,8
0,6
0,4
0,2
0
NaCl
EtOH
EtOH+LVVH7
LVVH7
1
2
3
4
5

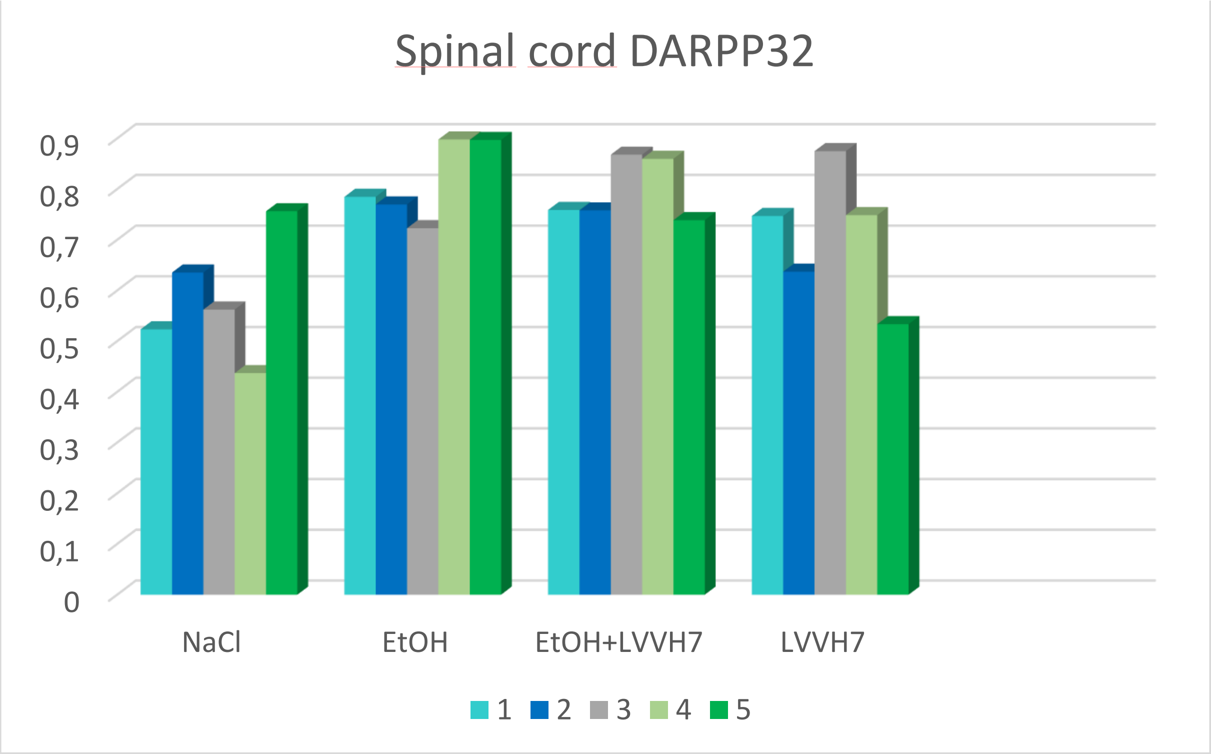

Figure S4. Immunassay of DARPP32 expression level as a result of EtOH administration and the effect of LVV-H7 co-administration.

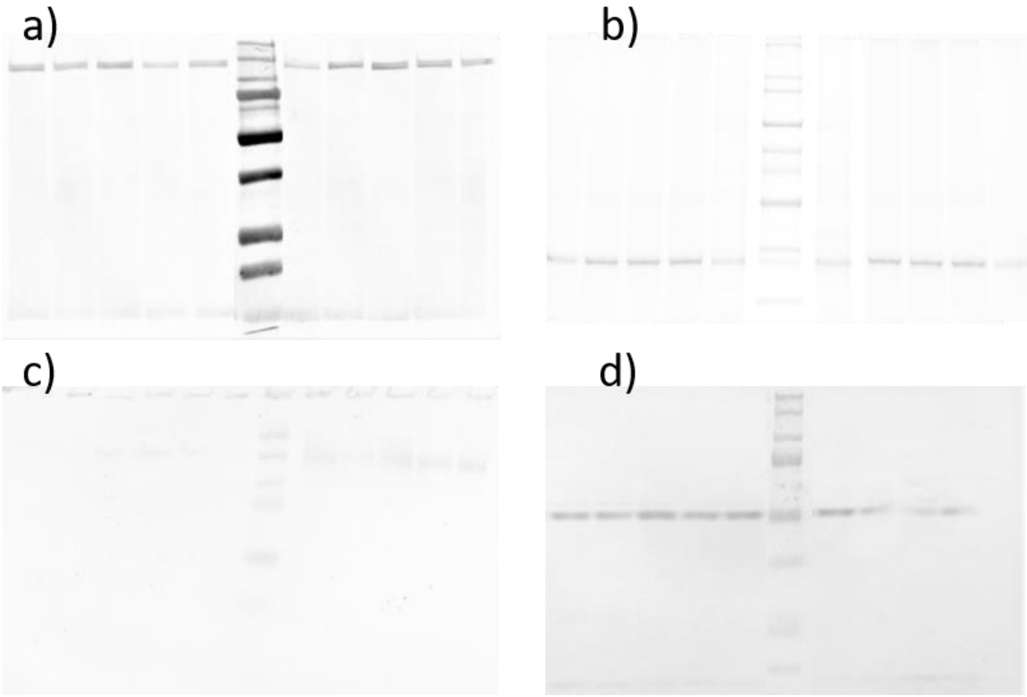

Figure S5. Exemplary scans of blots a) ACE1, b) DARP32, c) NMDA1, and d) OPRM1 in studied animal groups.

Table S1. Identified proteins bound to the LVV-H7 sequence, specific to the brain tissue in both group of rats treated with ethanol (Meta Score A) and the control group (Meta Score B).

Row	Accession	Name	Meta Score A	Meta Score B
1	STMN1_RAT	Stathmin OS=Rattus norvegicus GN=Stmn1 PE=1 SV=2	60.1	69.4
2	GNAO_RAT	Guanine nucleotide-binding protein G(o) subunit alpha OS=Rattus norvegicus GN=Gnao1 PE=1 SV=2	90.7	62.7
3	GPM6A_RAT	Neuronal membrane glycoprotein M6-a OS=Rattus norvegicus GN=Gpm6a PE=1 SV=1	42.5	57.5

4	SYN2_RAT	Synapsin-2 OS=Rattus norvegicus GN=Syn2 PE=1 SV=1	525.4	431.5
5	PRDX6_RAT	Peroxiredoxin-6 OS=Rattus norvegicus GN=Prdx6 PE=1 SV=3	35.5	0.0
6	GFAP_RAT	Glial fibrillary acidic protein OS=Rattus norvegicus GN=Gfap PE=1 SV=2	167.2	222.7
7	BACH_RAT	Cytosolic acyl coenzyme A thioester hydrolase OS=Rattus norvegicus GN=Acot7 PE=1 SV=4	49.6	45.3
8	MBP_RAT	Myelin basic protein OS=Rattus norvegicus GN=Mbp PE=1 SV=3	516.6	479.8
9	UCHL1_RAT	Ubiquitin carboxyl-terminal hydrolase isozyme L1 OS=Rattus norvegicus GN=Uchl1 PE=1 SV=2	98.9	109.2
10	SH3G2_RAT	Endophilin-A1 OS=Rattus norvegicus GN=Sh3gl2 PE=1 SV=2	0.0	46.4
11	SPYA_RAT	Serine--pyruvate aminotransferase, mitochondrial OS=Rattus norvegicus GN=Agxt PE=1 SV=1	20.3	0.0
12	MDHM_RAT	Malate dehydrogenase, mitochondrial OS=Rattus norvegicus GN=Mdh2 PE=1 SV=2	162.0	179.7
13	CISY_RAT	Citrate synthase, mitochondrial OS=Rattus norvegicus GN=Cs PE=1 SV=1	0.0	28.3
14	VAMP2_RAT	Vesicle-associated membrane protein 2 OS=Rattus norvegicus GN=Vamp2 PE=1 SV=2	120.8	113.6
15	RP3A_RAT	Rabphilin-3A OS=Rattus norvegicus GN=Rph3a PE=1 SV=1	124.6	128.8
16	PIF1_RAT	ATP-dependent DNA helicase PIF1 OS=Rattus norvegicus GN=Pif1 PE=2 SV=1	19.1	19.1
17	PP2BA_RAT	Serine/threonine-protein phosphatase 2B catalytic subunit alpha isoform OS=Rattus norvegicus GN=Ppp3ca PE=1 SV=1	80.2	39.1
18	EPHA6_RAT	Ephrin type-A receptor 6 OS=Rattus norvegicus GN=Epha6 PE=2 SV=2	18.2	0.0
19	NFH_RAT	Neurofilament heavy polypeptide OS=Rattus norvegicus GN=Nefh PE=1 SV=4	152.3	148.4
20	EF1A2_RAT	Elongation factor 1-alpha 2 OS=Rattus norvegicus GN=Eef1a2 PE=2 SV=1	117.2	140.6
21	AMPH_RAT	Amphiphysin OS=Rattus norvegicus GN=Amph PE=1 SV=1	74.1	0.0
22	VATB2_RAT	V-type proton ATPase subunit B, brain isoform OS=Rattus norvegicus GN=Atp6v1b2 PE=1 SV=1	227.0	222.2
23	TBB2A_RAT	Tubulin beta-2A chain OS=Rattus norvegicus GN=Tubb2a PE=1 SV=1	2646.2	2488.9
24	TPM3_RAT	Tropomyosin alpha-3 chain OS=Rattus norvegicus GN=Tpm3 PE=1 SV=2	155.0	146.0
25	ROA3_RAT	Heterogeneous nuclear ribonucleoprotein A3 OS=Rattus norvegicus GN=Hnrnpa3 PE=1 SV=1	186.1	194.1
26	TBB4B_RAT	Tubulin beta-4B chain OS=Rattus norvegicus GN=Tubb4b PE=1 SV=1	2326.1	2045.2
27	TBB2B_RAT	Tubulin beta-2B chain OS=Rattus norvegicus GN=Tubb2b PE=1 SV=1	2496.2	0.0
28	RAN_RAT	GTP-binding nuclear protein Ran OS=Rattus norvegicus GN=Ran PE=1 SV=3	0.0	18.1
29	CLAP2_RAT	CLIP-associating protein 2 OS=Rattus norvegicus GN=Clasp2 PE=2 SV=1	112.8	148.8
30	RAB12_RAT	Ras-related protein Rab-12 OS=Rattus norvegicus GN=Rab12 PE=1 SV=2	123.9	0.0
31	VIP_RAT	VIP peptides OS=Rattus norvegicus GN=Vip PE=1 SV=2	0.0	21.0
32	IDH3B_RAT	Isocitrate dehydrogenase [NAD] subunit beta, mitochondrial OS=Rattus norvegicus GN=Idh3B PE=1 SV=1	306.0	261.6
33	KHDR2_RAT	KH domain-containing, RNA-binding, signal transduction-associated protein 2 OS=Rattus norvegicus GN=Khdrbs2 PE=1 SV=1	21.0	0.0
34	VATE1_RAT	V-type proton ATPase subunit E 1 OS=Rattus norvegicus GN=Atp6v1e1 PE=1 SV=1	80.9	58.8
35	ACTC_RAT	Actin, alpha cardiac muscle 1 OS=Rattus norvegicus GN=Actc1 PE=2 SV=1	652.8	611.3
36	TBB3_RAT	Tubulin beta-3 chain OS=Rattus norvegicus GN=Tubb3 PE=1 SV=1	1952.8	1890.8
37	PCP4_RAT	Purkinje cell protein 4 OS=Rattus norvegicus GN=Pcp4 PE=1 SV=2	61.1	51.3
38	RN181_RAT	E3 ubiquitin-protein ligase RNF181 OS=Rattus norvegicus GN=Rnf181 PE=1 SV=1	24.7	0.0
39	DC1I1_RAT	Cytoplasmic dynein 1 intermediate chain 1 OS=Rattus norvegicus GN=Dync1i1 PE=1 SV=1	0.0	53.2
40	FAS_RAT	Fatty acid synthase OS=Rattus norvegicus GN=Fasn PE=1 SV=3	0.0	20.8
41	TBB5_RAT	Tubulin beta-5 chain OS=Rattus norvegicus GN=Tubb5 PE=1 SV=1	2583.7	2248.2
42	TRFE_RAT	Serotransferrin OS=Rattus norvegicus GN=Tf PE=1 SV=3	0.0	21.6
43	KCNH1_RAT	Potassium voltage-gated channel subfamily H member 1 OS=Rattus norvegicus GN=Kcnh1 PE=1 SV=1	20.0	0.0
44	AP2A2_RAT	AP-2 complex subunit alpha-2 OS=Rattus norvegicus GN=Ap2a2 PE=1 SV=3	168.5	141.0
45	PGK1_RAT	Phosphoglycerate kinase 1 OS=Rattus norvegicus GN=Pgk1 PE=1 SV=2	38.8	44.6
46	RAB3C_RAT	Ras-related protein Rab-3C OS=Rattus norvegicus GN=Rab3c PE=1 SV=1	152.7	137.5

47	CAND1_RAT	Cullin-associated NEDD8-dissociated protein 1 OS=Rattus norvegicus GN=Cand1 PE=1 SV=1	89.2	121.1
48	RAB10_RAT	Ras-related protein Rab-10 OS=Rattus norvegicus GN=Rab10 PE=1 SV=1	139.5	129.0
49	MYH10_RAT	Myosin-10 OS=Rattus norvegicus GN=Myh10 PE=2 SV=1	210.0	268.2
50	CALX_RAT	Calnexin OS=Rattus norvegicus GN=Canx PE=1 SV=1	21.4	42.3
51	K2C1_RAT	Keratin, type II cytoskeletal 1 OS=Rattus norvegicus GN=Krt1 PE=2 SV=1	80.6	85.0
52	DCE1_RAT	Glutamate decarboxylase 1 OS=Rattus norvegicus GN=Gad1 PE=2 SV=1	45.7	60.8
53	NPM_RAT	Nucleophosmin OS=Rattus norvegicus GN=Npm1 PE=1 SV=1	64.2	62.0
54	SCG1_RAT	Secretogranin-1 OS=Rattus norvegicus GN=Chgb PE=1 SV=2	0.0	35.4
55	TRIM3_RAT	Tripartite motif-containing protein 3 OS=Rattus norvegicus GN=Trim3 PE=1 SV=1	0.0	33.6
56	SYT1_RAT	Synaptotagmin-1 OS=Rattus norvegicus GN=Syt1 PE=1 SV=3	0.0	67.7
57	EF1A1_RAT	Elongation factor 1-alpha 1 OS=Rattus norvegicus GN=Eef1a1 PE=1 SV=1	85.4	0.0
58	KCTD5_RAT	BTB/POZ domain-containing protein KCTD5 OS=Rattus norvegicus GN=Kctd5 PE=2 SV=1	33.4	0.0
59	NEB1_RAT	Neurabin-1 OS=Rattus norvegicus GN=Ppp1r9a PE=1 SV=1	89.1	124.8
60	CH60_RAT	60 kDa heat shock protein, mitochondrial OS=Rattus norvegicus GN=Hspd1 PE=1 SV=1	291.1	305.6
61	ENOA_RAT	Alpha-enolase OS=Rattus norvegicus GN=Eno1 PE=1 SV=4	236.2	220.7
62	AP180_RAT	Clathrin coat assembly protein AP180 OS=Rattus norvegicus GN=Snap91 PE=1 SV=1	65.9	37.6
63	TKT_RAT	Transketolase OS=Rattus norvegicus GN=Tkt PE=1 SV=1	85.8	68.8
64	H2AZ_RAT	Histone H2A.Z OS=Rattus norvegicus GN=H2afz PE=1 SV=2	43.0	72.9
65	DYN1_RAT	Dynamin-1 OS=Rattus norvegicus GN=Dnm1 PE=1 SV=2	504.3	691.0
66	PGAM1_RAT	Phosphoglycerate mutase 1 OS=Rattus norvegicus GN=Pgam1 PE=1 SV=4	243.3	181.8
67	MARCS_RAT	Myristoylated alanine-rich C-kinase substrate OS=Rattus norvegicus GN=Marcks PE=1 SV=2	402.8	418.6
68	SHLB2_RAT	Endophilin-B2 OS=Rattus norvegicus GN=Sh3glb2 PE=2 SV=2	27.8	0.0
69	HS90A_RAT	Heat shock protein HSP 90-alpha OS=Rattus norvegicus GN=Hsp90aa1 PE=1 SV=3	144.6	129.5
70	DNM1L_RAT	Dynamin-1-like protein OS=Rattus norvegicus GN=Dnm1l PE=1 SV=1	764.3	985.8
71	KCRB_RAT	Creatine kinase B-type OS=Rattus norvegicus GN=Ckb PE=1 SV=2	582.6	549.0
72	AINX_RAT	Alpha-internexin OS=Rattus norvegicus GN=Ina PE=1 SV=2	465.1	558.7
73	G6PI_RAT	Glucose-6-phosphate isomerase OS=Rattus norvegicus GN=Gpi PE=1 SV=1	42.2	39.4
74	LDHB_RAT	L-lactate dehydrogenase B chain OS=Rattus norvegicus GN=Ldhb PE=1 SV=2	45.8	71.3
75	ARF3_RAT	ADP-ribosylation factor 3 OS=Rattus norvegicus GN=Arf3 PE=1 SV=2	78.0	0.0
76	HS90B_RAT	Heat shock protein HSP 90-beta OS=Rattus norvegicus GN=Hsp90ab1 PE=1 SV=4	120.6	0.0
77	APOA2_RAT	Apolipoprotein A-II OS=Rattus norvegicus GN=Apoa2 PE=2 SV=1	0.0	37.0
78	AT1B1_RAT	Sodium/potassium-transporting ATPase subunit beta-1 OS=Rattus norvegicus GN=Atp1b1 PE=1 SV=1	63.0	60.6
79	CN37_RAT	2',3'-cyclic-nucleotide 3'-phosphodiesterase OS=Rattus norvegicus GN=Cnp PE=1 SV=2	304.2	390.3
80	AAK1_RAT	AP2-associated protein kinase 1 OS=Rattus norvegicus GN=Aak1 PE=1 SV=1	0.0	57.6
81	ROA2_RAT	Heterogeneous nuclear ribonucleoproteins A2/B1 OS=Rattus norvegicus GN=Hnrnpa2b1 PE=1 SV=1	304.5	298.7
82	DREB_RAT	Drebrin OS=Rattus norvegicus GN=Dbn1 PE=1 SV=3	381.1	467.3
83	LPHN2_RAT	Latrophilin-2 OS=Rattus norvegicus GN=Lphn2 PE=2 SV=2	22.4	0.0
84	G3P_RAT	Glyceraldehyde-3-phosphate dehydrogenase OS=Rattus norvegicus GN=Gapdh PE=1 SV=3	244.9	275.9
85	RAF1_RAT	RAF proto-oncogene serine/threonine-protein kinase OS=Rattus norvegicus GN=Raf1 PE=1 SV=1	0.0	23.5
86	DYN3_RAT	Dynamin-3 OS=Rattus norvegicus GN=Dnm3 PE=1 SV=2	0.0	214.8
87	RBM43_RAT	RNA-binding protein 43 OS=Rattus norvegicus GN=Rbm43 PE=2 SV=1	0.0	18.7
88	RTN3_RAT	Reticulon-3 OS=Rattus norvegicus GN=Rtn3 PE=1 SV=1	53.6	45.0
89	CPNS1_RAT	Calpain small subunit 1 OS=Rattus norvegicus GN=Capns1 PE=1 SV=3	77.3	95.2
90	RD23B_RAT	UV excision repair protein RAD23 homolog B OS=Rattus norvegicus GN=Rad23b PE=1 SV=1	49.9	57.1
91	TCPA_RAT	T-complex protein 1 subunit alpha OS=Rattus norvegicus GN=Tcp1 PE=1 SV=1	22.8	0.0
92	CKD18_RAT	Cyclin-dependent kinase 18 OS=Rattus norvegicus GN=Cdk18 PE=1 SV=1	33.9	0.0

93	PYGB_RAT	Glycogen phosphorylase, brain form (Fragment) OS=Rattus norvegicus GN=Pygb PE=1 SV=3	172.3	133.3
94	MP2K1_RAT	Dual specificity mitogen-activated protein kinase kinase 1 OS=Rattus norvegicus GN=Map2k1 PE=1 SV=2	108.9	197.4
95	K1C10_RAT	Keratin, type I cytoskeletal 10 OS=Rattus norvegicus GN=Krt10 PE=3 SV=1	40.7	25.5
96	AATC_RAT	Aspartate aminotransferase, cytoplasmic OS=Rattus norvegicus GN=Got1 PE=1 SV=3	195.6	181.4
97	GABT_RAT	4-aminobutyrate aminotransferase, mitochondrial OS=Rattus norvegicus GN=Abat PE=1 SV=3	170.3	177.2
98	ERR1_RAT	Steroid hormone receptor ERR1 OS=Rattus norvegicus GN=Esrra PE=2 SV=2	0.0	33.8
99	NCDN_RAT	Neurochondrin OS=Rattus norvegicus GN=Ncdn PE=1 SV=2	143.1	116.4
100	CNTN1_RAT	Contactin-1 OS=Rattus norvegicus GN=Cntn1 PE=1 SV=2	168.3	57.1
101	VISL1_RAT	Visinin-like protein 1 OS=Rattus norvegicus GN=Vsnl1 PE=1 SV=2	42.5	0.0
102	PPIA_RAT	Peptidyl-prolyl cis-trans isomerase A OS=Rattus norvegicus GN=Ppia PE=1 SV=2	122.5	134.8
103	LACE1_RAT	Lactation elevated protein 1 OS=Rattus norvegicus GN=Lace1 PE=2 SV=1	18.8	0.0
104	RCN2_RAT	Reticulocalbin-2 OS=Rattus norvegicus GN=Rcn2 PE=1 SV=2	120.2	0.0
105	CAP1_RAT	Adenylyl cyclase-associated protein 1 OS=Rattus norvegicus GN=Cap1 PE=1 SV=3	103.8	112.1
106	CAZA2_RAT	F-actin-capping protein subunit alpha-2 OS=Rattus norvegicus GN=Capza2 PE=1 SV=1	106.7	74.4
107	1433G_RAT	14-3-3 protein gamma OS=Rattus norvegicus GN=Ywhag PE=1 SV=2	130.5	109.3
108	ALDOA_RAT	Fructose-bisphosphate aldolase A OS=Rattus norvegicus GN=Aldoa PE=1 SV=2	307.7	399.4
109	1433T_RAT	14-3-3 protein theta OS=Rattus norvegicus GN=Ywhaq PE=1 SV=1	139.6	123.1
110	1433Z_RAT	14-3-3 protein zeta/delta OS=Rattus norvegicus GN=Ywhaz PE=1 SV=1	218.3	137.5
111	MAP6_RAT	Microtubule-associated protein 6 OS=Rattus norvegicus GN=Map6 PE=1 SV=1	489.3	511.8
112	IF4A2_RAT	Eukaryotic initiation factor 4A-II OS=Rattus norvegicus GN=Eif4a2 PE=1 SV=1	37.2	58.5
113	1433F_RAT	14-3-3 protein eta OS=Rattus norvegicus GN=Ywhah PE=1 SV=2	103.8	82.8
114	1433B_RAT	14-3-3 protein beta/alpha OS=Rattus norvegicus GN=Ywhab PE=1 SV=3	142.8	142.8
115	HYOU1_RAT	Hypoxia up-regulated protein 1 OS=Rattus norvegicus GN=Hyou1 PE=1 SV=1	0.0	19.3
116	KCC4_RAT	Calcium/calmodulin-dependent protein kinase type IV OS=Rattus norvegicus GN=Camk4 PE=1 SV=3	192.3	160.7
117	ATPB_RAT	ATP synthase subunit beta, mitochondrial OS=Rattus norvegicus GN=Atp5b PE=1 SV=2	80.3	0.0
118	NECP1_RAT	Adaptin ear-binding coat-associated protein 1 OS=Rattus norvegicus GN=Necap1 PE=1 SV=1	34.2	0.0
119	ASGR1_RAT	Asialoglycoprotein receptor 1 OS=Rattus norvegicus GN=Asgr1 PE=1 SV=2	20.8	0.0
120	HBB2_RAT	Hemoglobin subunit beta-2 OS=Rattus norvegicus PE=1 SV=2	382.8	488.8
121	CXA1_RAT	Gap junction alpha-1 protein OS=Rattus norvegicus GN=Gja1 PE=1 SV=2	59.4	61.6
122	CPLX1_RAT	Complexin-1 OS=Rattus norvegicus GN=Cplx1 PE=1 SV=1	136.2	118.4
123	COLI_RAT	Pro-opiomelanocortin OS=Rattus norvegicus GN=Pomc PE=1 SV=1	110.5	125.3
124	STXB1_RAT	Syntaxin-binding protein 1 OS=Rattus norvegicus GN=Stxbp1 PE=1 SV=1	99.8	123.4
125	SPTN1_RAT	Spectrin alpha chain, non-erythrocytic 1 OS=Rattus norvegicus GN=Sptan1 PE=1 SV=2	2446.9	3797.8
126	SYN1_RAT	Synapsin-1 OS=Rattus norvegicus GN=Syn1 PE=1 SV=3	673.0	535.6
127	DCTN2_RAT	Dynactin subunit 2 OS=Rattus norvegicus GN=Dctn2 PE=1 SV=1	57.6	50.6
128	CLH1_RAT	Clathrin heavy chain 1 OS=Rattus norvegicus GN=Cltc PE=1 SV=3	491.1	554.1
129	VIME_RAT	Vimentin OS=Rattus norvegicus GN=Vim PE=1 SV=2	33.4	26.5
130	MYH9_RAT	Myosin-9 OS=Rattus norvegicus GN=Myh9 PE=1 SV=3	60.8	0.0
131	RL40_RAT	Ubiquitin-60S ribosomal protein L40 OS=Rattus norvegicus GN=Uba52 PE=1 SV=2	29.3	44.6
132	TPIS_RAT	Triosephosphate isomerase OS=Rattus norvegicus GN=Tpi1 PE=1 SV=2	152.8	127.3
133	SYNJ1_RAT	Synaptojanin-1 OS=Rattus norvegicus GN=Synj1 PE=1 SV=3	21.0	73.0
134	CPLX2_RAT	Complexin-2 OS=Rattus norvegicus GN=Cplx2 PE=1 SV=1	76.7	75.7
135	NSRP1_RAT	Nuclear speckle splicing regulatory protein 1 OS=Rattus norvegicus GN=Nsrp1 PE=2 SV=1	0.0	21.2
136	H2B1_RAT	Histone H2B type 1 OS=Rattus norvegicus PE=1 SV=2	61.9	114.4
137	HSP7C_RAT	Heat shock cognate 71 kDa protein OS=Rattus norvegicus GN=Hspa8 PE=1 SV=1	1040.5	954.3

138	NSF_RAT	Vesicle-fusing ATPase OS=Rattus norvegicus GN=Nsf PE=1 SV=1	370.6	405.0
139	EF2_RAT	Elongation factor 2 OS=Rattus norvegicus GN=Eef2 PE=1 SV=4	90.5	48.2
140	HBB1_RAT	Hemoglobin subunit beta-1 OS=Rattus norvegicus GN=Hbb PE=1 SV=3	545.2	638.0
141	UBQL1_RAT	Ubiquilin-1 OS=Rattus norvegicus GN=Ubqln1 PE=1 SV=1	20.2	33.2
142	CYTC_RAT	Cystatin-C OS=Rattus norvegicus GN=Cst3 PE=1 SV=2	61.1	54.0
143	KCC2A_RAT	Calcium/calmodulin-dependent protein kinase type II subunit alpha OS=Rattus norvegicus GN=Camk2a PE=1 SV=1	39.9	38.6
144	ADDA_RAT	Alpha-adducin OS=Rattus norvegicus GN=Add1 PE=2 SV=2	24.9	91.9
145	VGF_RAT	Neurosecretory protein VGF OS=Rattus norvegicus GN=Vgf PE=1 SV=3	33.4	25.7
146	SCG2_RAT	Secretogranin-2 OS=Rattus norvegicus GN=Scg2 PE=1 SV=1	192.0	41.9
147	TBA4A_RAT	Tubulin alpha-4A chain OS=Rattus norvegicus GN=Tuba4a PE=2 SV=1	1514.2	1569.0
148	RRP8_RAT	Ribosomal RNA-processing protein 8 OS=Rattus norvegicus GN=Rrp8 PE=2 SV=1	37.0	34.0
149	AP2B1_RAT	AP-2 complex subunit beta OS=Rattus norvegicus GN=Ap2b1 PE=1 SV=1	101.0	109.4
150	SPTN2_RAT	Spectrin beta chain, non-erythrocytic 2 OS=Rattus norvegicus GN=Sptbn2 PE=1 SV=2	1809.9	2154.3
151	EFHD2_RAT	EF-hand domain-containing protein D2 OS=Rattus norvegicus GN=Efhd2 PE=1 SV=1	49.8	19.8
152	S100B_RAT	Protein S100-B OS=Rattus norvegicus GN=S100b PE=1 SV=2	172.1	189.6
153	ACTG_RAT	Actin, cytoplasmic 2 OS=Rattus norvegicus GN=Actg1 PE=1 SV=1	1596.6	1524.6
154	AT1A3_RAT	Sodium/potassium-transporting ATPase subunit alpha-3 OS=Rattus norvegicus GN=Atp1a3 PE=1 SV=2	474.9	548.1
155	FKB1A_RAT	Peptidyl-prolyl cis-trans isomerase FKBP1A OS=Rattus norvegicus GN=Fkbp1a PE=1 SV=3	84.9	92.5
156	GDIA_RAT	Rab GDP dissociation inhibitor alpha OS=Rattus norvegicus GN=Gdi1 PE=1 SV=1	372.7	196.1
157	STIP1_RAT	Stress-induced-phosphoprotein 1 OS=Rattus norvegicus GN=Stip1 PE=1 SV=1	38.3	36.4
158	NCAM1_RAT	Neural cell adhesion molecule 1 OS=Rattus norvegicus GN=Ncam1 PE=1 SV=1	102.3	176.7
159	IP3KA_RAT	Inositol-trisphosphate 3-kinase A OS=Rattus norvegicus GN=Itpka PE=1 SV=3	142.8	106.1
160	KPYM_RAT	Pyruvate kinase PKM OS=Rattus norvegicus GN=Pkm PE=1 SV=3	535.9	383.5
161	APOE_RAT	Apolipoprotein E OS=Rattus norvegicus GN=Apoe PE=1 SV=2	101.6	126.0
162	MPP4_RAT	MAGUK p55 subfamily member 4 OS=Rattus norvegicus GN=Mpp4 PE=1 SV=1	0.0	20.5
163	PACN1_RAT	Protein kinase C and casein kinase substrate in neurons protein 1 OS=Rattus norvegicus GN=Pacsin1 PE=1 SV=1	0.0	19.6
164	DCE2_RAT	Glutamate decarboxylase 2 OS=Rattus norvegicus GN=Gad2 PE=1 SV=1	40.7	81.9
165	PURB_RAT	Transcriptional activator protein Pur-beta OS=Rattus norvegicus GN=Purb PE=1 SV=3	57.6	75.5
166	EAA1_RAT	Excitatory amino acid transporter 1 OS=Rattus norvegicus GN=Slc1a3 PE=1 SV=2	60.8	0.0
167	RAB7A_RAT	Ras-related protein Rab-7a OS=Rattus norvegicus GN=Rab7a PE=1 SV=2	85.8	51.3
168	MINK1_RAT	Misshapen-like kinase 1 OS=Rattus norvegicus GN=Mink1 PE=1 SV=2	0.0	40.2
169	MATR3_RAT	Matrin-3 OS=Rattus norvegicus GN=Matr3 PE=1 SV=2	182.2	225.7
170	SYUA_RAT	Alpha-synuclein OS=Rattus norvegicus GN=Snca PE=1 SV=1	50.3	0.0
171	ABCF1_RAT	ATP-binding cassette sub-family F member 1 OS=Rattus norvegicus GN=Abcf1 PE=1 SV=1	21.3	21.2
172	TRY1_RAT	Anionic trypsin-1 OS=Rattus norvegicus GN=Prss1 PE=1 SV=1	72.2	74.1
173	NFM_RAT	Neurofilament medium polypeptide OS=Rattus norvegicus GN=Nefm PE=1 SV=4	526.4	582.0
174	ACON_RAT	Aconitate hydratase, mitochondrial OS=Rattus norvegicus GN=Aco2 PE=1 SV=2	127.4	123.6
175	ODP2_RAT	Dihydrolipoyllysine-residue acetyltransferase component of pyruvate dehydrogenase complex, mitochondrial OS=Rattus norvegicus GN=Dlat PE=1 SV=3	26.9	0.0
176	ASPD_RAT	Putative L-aspartate dehydrogenase OS=Rattus norvegicus GN=Aspdh PE=2 SV=1	18.8	0.0
177	SCOC_RAT	Short coiled-coil protein OS=Rattus norvegicus GN=Scoc PE=2 SV=1	43.2	36.7
178	ATPA_RAT	ATP synthase subunit alpha, mitochondrial OS=Rattus norvegicus GN=Atp5a1 PE=1 SV=2	202.3	92.3

179	GSTA3_RAT	Glutathione S-transferase alpha-3 OS=Rattus norvegicus GN=Gsta3 PE=1 SV=3	46.8	22.5
180	ALDOC_RAT	Fructose-bisphosphate aldolase C OS=Rattus norvegicus GN=Aldoc PE=1 SV=3	525.3	477.9
181	HNRPF_RAT	Heterogeneous nuclear ribonucleoprotein F OS=Rattus norvegicus GN=Hnrnpf PE=1 SV=3	39.1	41.7
182	NDKA_RAT	Nucleoside diphosphate kinase A OS=Rattus norvegicus GN=Nme1 PE=1 SV=1	28.1	87.2
183	VATC1_RAT	V-type proton ATPase subunit C 1 OS=Rattus norvegicus GN=Atp6v1c1 PE=2 SV=1	60.4	84.3
184	FUBP2_RAT	Far upstream element-binding protein 2 OS=Rattus norvegicus GN=Khsrp PE=1 SV=1	63.0	60.8
185	NEB2_RAT	Neurabin-2 OS=Rattus norvegicus GN=Ppp1r9b PE=1 SV=1	102.8	150.9
186	PGM1_RAT	Phosphoglucomutase-1 OS=Rattus norvegicus GN=Pgm1 PE=1 SV=2	55.0	33.1
187	REM2_RAT	GTP-binding protein REM 2 OS=Rattus norvegicus GN=Rem2 PE=1 SV=2	0.0	32.7
188	IMB1_RAT	Importin subunit beta-1 OS=Rattus norvegicus GN=Kpnb1 PE=1 SV=1	50.1	55.8
189	TRIM9_RAT	E3 ubiquitin-protein ligase TRIM9 OS=Rattus norvegicus GN=Trim9 PE=1 SV=1	107.6	48.2
190	MYO5A_RAT	Unconventional myosin-Va OS=Rattus norvegicus GN=Myo5a PE=1 SV=1	115.0	48.4
191	THY1_RAT	Thy-1 membrane glycoprotein OS=Rattus norvegicus GN=Thy1 PE=1 SV=1	78.3	68.6
192	ATX10_RAT	Ataxin-10 OS=Rattus norvegicus GN=Atxn10 PE=1 SV=1	55.4	71.6
193	RUFY3_RAT	Protein RUFY3 OS=Rattus norvegicus GN=Rufy3 PE=1 SV=1	87.2	104.4
194	AT2A2_RAT	Sarcoplasmic/endoplasmic reticulum calcium ATPase 2 OS=Rattus norvegicus GN=Atp2a2 PE=1 SV=1	0.0	136.9
195	DYHC1_RAT	Cytoplasmic dynein 1 heavy chain 1 OS=Rattus norvegicus GN=Dync1h1 PE=1 SV=1	568.7	548.0
196	SH3G1_RAT	Endophilin-A2 OS=Rattus norvegicus GN=Sh3gl1 PE=1 SV=1	60.2	0.0
197	ACTN1_RAT	Alpha-actinin-1 OS=Rattus norvegicus GN=Actn1 PE=1 SV=1	293.5	324.4
198	MTAP2_RAT	Microtubule-associated protein 2 OS=Rattus norvegicus GN=Map2 PE=1 SV=3	585.6	401.9
199	RPA1_RAT	DNA-directed RNA polymerase I subunit RPA1 OS=Rattus norvegicus GN=Polr1a PE=1 SV=1	0.0	19.5
200	ALBU_RAT	Serum albumin OS=Rattus norvegicus GN=Alb PE=1 SV=2	306.0	186.1
201	RAP1B_RAT	Ras-related protein Rap-1b OS=Rattus norvegicus GN=Rap1b PE=2 SV=2	37.5	50.6
202	CRIP2_RAT	Cysteine-rich protein 2 OS=Rattus norvegicus GN=Crip2 PE=2 SV=1	67.8	0.0
203	HBA_RAT	Hemoglobin subunit alpha-1/2 OS=Rattus norvegicus GN=Hba1 PE=1 SV=3	247.4	230.3
204	HSP74_RAT	Heat shock 70 kDa protein 4 OS=Rattus norvegicus GN=Hspa4 PE=1 SV=1	155.2	113.4
205	PROF2_RAT	Profilin-2 OS=Rattus norvegicus GN=Pfn2 PE=1 SV=3	60.6	47.7
206	DPYL2_RAT	Dihydropyrimidinase-related protein 2 OS=Rattus norvegicus GN=Dpysl2 PE=1 SV=1	882.5	680.8
207	CALM_RAT	Calmodulin OS=Rattus norvegicus GN=Calm1 PE=1 SV=2	330.5	413.9
208	OBP_RAT	Odorant-binding protein OS=Rattus norvegicus GN=Obp1f PE=1 SV=1	0.0	23.8
209	RAB2A_RAT	Ras-related protein Rab-2A OS=Rattus norvegicus GN=Rab2a PE=1 SV=1	38.3	37.1
210	GLSK_RAT	Glutaminase kidney isoform, mitochondrial OS=Rattus norvegicus GN=Gls PE=1 SV=2	32.6	0.0
211	NFL_RAT	Neurofilament light polypeptide OS=Rattus norvegicus GN=Nefl PE=1 SV=3	108.8	143.3
212	KCNH6_RAT	Potassium voltage-gated channel subfamily H member 6 OS=Rattus norvegicus GN=Kcnh6 PE=1 SV=1	26.3	0.0
213	SNP25_RAT	Synaptosomal-associated protein 25 OS=Rattus norvegicus GN=Snap25 PE=1 SV=1	211.9	241.0
214	VAMP1_RAT	Vesicle-associated membrane protein 1 OS=Rattus norvegicus GN=Vamp1 PE=1 SV=2	65.8	0.0
215	GSTM1_RAT	Glutathione S-transferase Mu 1 OS=Rattus norvegicus GN=Gstm1 PE=1 SV=2	28.3	29.0
216	PRDX5_RAT	Peroxiredoxin-5, mitochondrial OS=Rattus norvegicus GN=Prdx5 PE=1 SV=1	93.8	83.3
217	PARK7_RAT	Protein deglycase DJ-1 OS=Rattus norvegicus GN=Park7 PE=1 SV=1	57.6	64.6
218	RTN1_RAT	Reticulon-1 OS=Rattus norvegicus GN=Rtn1 PE=2 SV=1	30.7	48.7
219	ROA1_RAT	Heterogeneous nuclear ribonucleoprotein A1 OS=Rattus norvegicus GN=Hnrnpa1 PE=1 SV=3	101.4	64.4
220	SIR2_RAT	NAD-dependent protein deacetylase sirtuin-2 OS=Rattus norvegicus GN=Sirt2 PE=1 SV=1	178.8	177.0
221	ALDR_RAT	Aldose reductase OS=Rattus norvegicus GN=Akr1b1 PE=1 SV=3	42.7	0.0
222	GRP75_RAT	Stress-70 protein, mitochondrial OS=Rattus norvegicus GN=Hspa9 PE=1 SV=3	23.9	0.0

223	IDH3A_RAT	Isocitrate dehydrogenase [NAD] subunit alpha, mitochondrial OS=Rattus norvegicus GN=Idh3a PE=1 SV=1	96.9	28.2
224	CNGA4_RAT	Cyclic nucleotide-gated cation channel alpha-4 OS=Rattus norvegicus GN=Cnga4 PE=2 SV=1	0.0	22.3
225	TCPG_RAT	T-complex protein 1 subunit gamma OS=Rattus norvegicus GN=Cct3 PE=1 SV=1	18.7	0.0
226	CAD23_RAT	Cadherin-23 OS=Rattus norvegicus GN=Cdh23 PE=2 SV=1	28.3	38.8
227	ENOG_RAT	Gamma-enolase OS=Rattus norvegicus GN=Eno2 PE=1 SV=2	240.7	186.5
228	ACTZ_RAT	Alpha-centractin OS=Rattus norvegicus GN=Actr1a PE=1 SV=1	160.2	138.9
229	TBA1B_RAT	Tubulin alpha-1B chain OS=Rattus norvegicus GN=Tuba1b PE=1 SV=1	1902.9	1913.3
230	PURA_RAT	Transcriptional activator protein Pur-alpha (Fragments) OS=Rattus norvegicus GN=Pura PE=1 SV=1	150.3	138.6
231	GLNA_RAT	Glutamine synthetase OS=Rattus norvegicus GN=Glul PE=1 SV=3	249.2	166.8
232	HCD2_RAT	3-hydroxyacyl-CoA dehydrogenase type-2 OS=Rattus norvegicus GN=Hsd17b10 PE=1 SV=3	70.5	33.6
233	TBA1A_RAT	Tubulin alpha-1A chain OS=Rattus norvegicus GN=Tuba1a PE=1 SV=1	1916.3	1939.6
234	SYUB_RAT	Beta-synuclein OS=Rattus norvegicus GN=Sncb PE=1 SV=1	134.0	133.0
235	IDHG1_RAT	Isocitrate dehydrogenase [NAD] subunit gamma 1, mitochondrial OS=Rattus norvegicus GN=Idh3g PE=2 SV=2	204.8	147.0
236	TAGL3_RAT	Transgelin-3 OS=Rattus norvegicus GN=Tagln3 PE=1 SV=2	269.7	349.8
237	AP3M2_RAT	AP-3 complex subunit mu-2 OS=Rattus norvegicus GN=Ap3m2 PE=2 SV=1	22.2	0.0
238	DPYL1_RAT	Dihydropyrimidinase-related protein 1 OS=Rattus norvegicus GN=Crmp1 PE=1 SV=1	180.5	165.0
239	MDHC_RAT	Malate dehydrogenase, cytoplasmic OS=Rattus norvegicus GN=Mdh1 PE=1 SV=3	134.1	171.6
240	AP2M1_RAT	AP-2 complex subunit mu OS=Rattus norvegicus GN=Ap2m1 PE=1 SV=1	47.7	37.3
241	GSK3B_RAT	Glycogen synthase kinase-3 beta OS=Rattus norvegicus GN=Gsk3b PE=1 SV=1	0.0	20.1
242	RIN1_RAT	Ras and Rab interactor 1 OS=Rattus norvegicus GN=Rin1 PE=2 SV=2	67.4	33.6
243	UBA1_RAT	Ubiquitin-like modifier-activating enzyme 1 OS=Rattus norvegicus GN=Uba1 PE=1 SV=1	0.0	34.6
244	SYN3_RAT	Synapsin-3 OS=Rattus norvegicus GN=Syn3 PE=1 SV=1	106.2	105.5
245	EFTU_RAT	Elongation factor Tu, mitochondrial OS=Rattus norvegicus GN=Tufm PE=1 SV=1	220.0	183.9
246	KPCB_RAT	Protein kinase C beta type OS=Rattus norvegicus GN=Prkcb PE=1 SV=3	66.8	119.2
247	MAP1A_RAT	Microtubule-associated protein 1A OS=Rattus norvegicus GN=Map1a PE=1 SV=1	117.8	144.3
248	CELF2_RAT	CUGBP Elav-like family member 2 OS=Rattus norvegicus GN=Celf2 PE=2 SV=1	87.0	93.4
249	ADDB_RAT	Beta-adducin OS=Rattus norvegicus GN=Add2 PE=2 SV=4	36.6	28.4
250	KAD1_RAT	Adenylate kinase isoenzyme 1 OS=Rattus norvegicus GN=Ak1 PE=1 SV=3	180.6	170.9
251	PPR1B_RAT	Protein phosphatase 1 regulatory subunit 1B OS=Rattus norvegicus GN=Ppp1r1b PE=2 SV=1	67.2	31.5
252	PDXK_RAT	Pyridoxal kinase OS=Rattus norvegicus GN=Pdxk PE=1 SV=1	148.2	103.5
253	1433E_RAT	14-3-3 protein epsilon OS=Rattus norvegicus GN=Ywhae PE=1 SV=1	90.7	95.3
254	TRY3_RAT	Cationic trypsin-3 OS=Rattus norvegicus GN=Try3 PE=2 SV=1	0.0	33.0
255	YKT6_RAT	Synaptobrevin homolog YKT6 OS=Rattus norvegicus GN=Ykt6 PE=1 SV=1	35.1	185.4
256	RB11A_RAT	Ras-related protein Rab-11A OS=Rattus norvegicus GN=Rab11a PE=1 SV=3	92.0	45.7
257	WEE1_RAT	Wee1-like protein kinase OS=Rattus norvegicus GN=Wee1 PE=2 SV=1	0.0	21.3

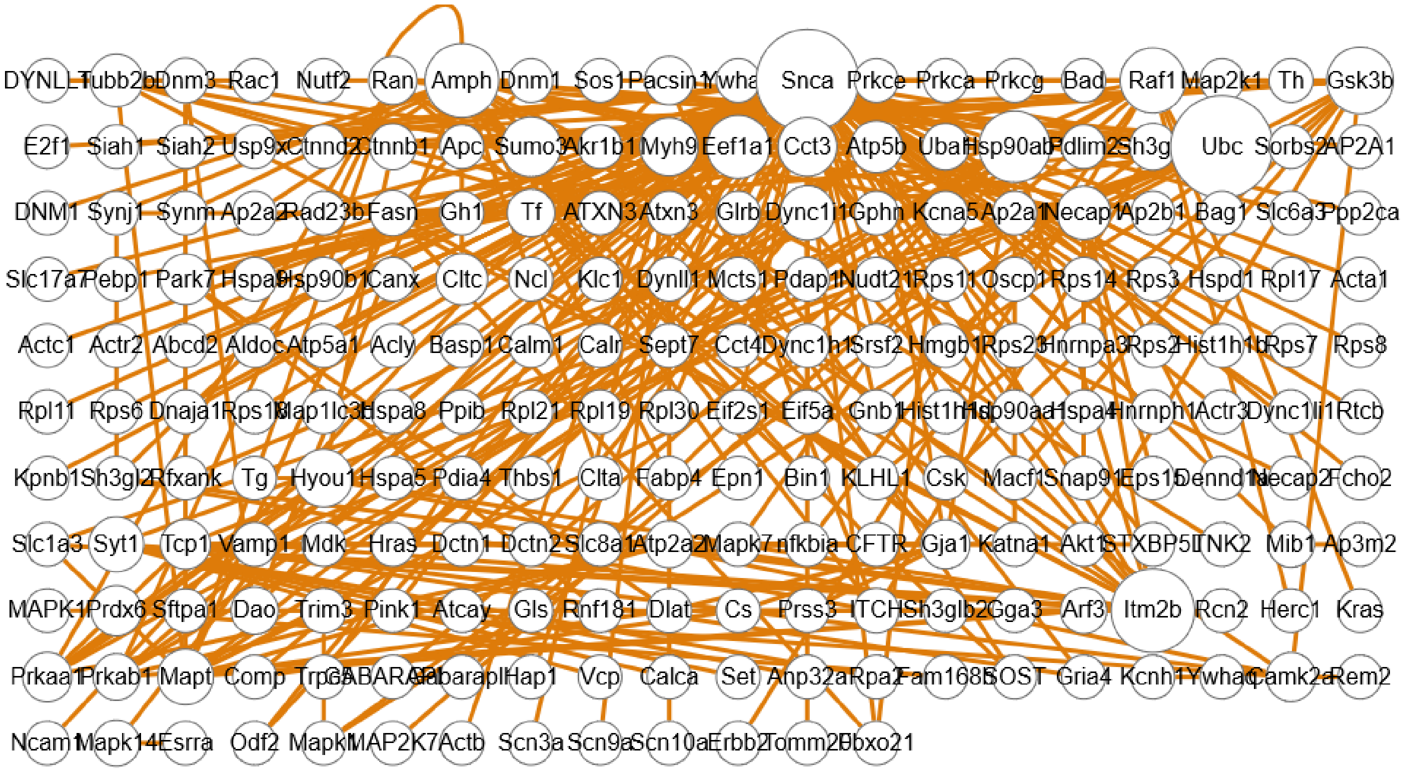

Figure S6. Genetic (green) and physical (brown) interaction networks between regulated proteins' binding to the LVV-H7 sequence characteristic of the brain tissue in both group of rats treated with ethanol and the control group with all other genes in the database. Interactions established by the easyN from bioGRID database (Human interactions).
